# Efficient DNA–Polymer Coupling in Organic Solvents: A Survey of Amide Coupling, Thiol-Ene and Tetrazine–Norbornene Chemistries Applied to Conjugation of Poly(*N*-Isopropylacrylamide)

**DOI:** 10.1038/srep39192

**Published:** 2016-12-16

**Authors:** Thomas R. Wilks, Rachel K. O’Reilly

**Affiliations:** 1 University of Warwick, Department of Chemistry, Coventry, CV4 7AL, UK

## Abstract

A range of chemistries were explored for the efficient covalent conjugation of DNA to poly(*N*-isopropylacrylamide) (poly(NIPAM)) in organic solvents. Amide coupling and thiol–ene Michael addition were found to be ineffective for the synthesis of the desired products. However, the inverse electron-demand Diels–Alder (DA_*inv*_) reaction between tetrazine (Tz) and norbornene (Nb) was found to give DNA–polymer conjugates in good yields (up to 40%) in organic solvents (*N,N*-dimethylformamide, *N,N*-dimethylacetamide and *N*-methyl-2-pyrrolidone), and without the need for a catalyst. Methods for the synthesis of Tz-and Nb- functionalised DNA were developed, along with a post-polymerisation functionalisation strategy for the production of Tz-functionalised polymers.

Since the introduction by Seeman, Rothemund and others of the concept of DNA origami, the use of nucleic acids in nanoscale materials has blossomed^1^,^2^. DNA’s unique ability to form perfectly-controlled higher order structures makes it very appealing to materials scientists. Highly complex objects can now be fabricated on the nanoscale, including a lockable DNA box, structures with three dimensional curvature, tetrahedra, prisms and cubes^3^,^4^,^5^,^6^,^7^. It is difficult to envisage how these structures could have been formed by any other currently available technology.

The utility of DNA can be further enhanced by direct conjugation to synthetic polymers, which bring an added dimension in terms of their solvophilicity, functionality and potential to respond to external stimuli. For example, Mirkin *et al*. attached poly(styrene) (PS) to a short segment of single stranded DNA (ssDNA)^8^. Upon resuspension in water the PS-ssDNA amphiphile assembled into well-defined micelles. The ssDNA located in the corona of the micelle was then used as a functional handle - in this case, hybrid materials were created when the micelles were mixed with gold nanoparticles bearing the complementary DNA strand through the formation of a DNA duplex. Herrmann *et al*. used a similar approach to form poly(propylene oxide) (PPO)-ssDNA micelles, and these were used to perform DNA-templated synthesis^9^, synergistically deliver anti-cancer therapeutics^10^, and to create structures whose morphology could be tuned by the addition of different ssDNA strands^11^. Gianneschi *et al*. also exploited the use of DNA hybridisation in combination with enzymatic tools to create ‘programmable shape-shifting micelles’^12^. The elegant work of Sleiman *et al*. further demonstrates the unique properties that DNA-polymer conjugates can possess. They used DNA to control the precise placement of polymer domains within hybrid structures, and demonstrated the ability of polymers conjugated to DNA to form discrete nanocube structures, which predictably changed the polymer’s self-assembly behaviour, as well as introducing a new driving force for the assembly of DNA nanostructures in the form of hydrophobic interactions^13^,^14^,^15^,^16^. Our own group also made the first report of a DNA-polymer conjugate in which the DNA was used to form part of a discrete nanostructure, in this case a DNA tetrahedron^17^. The polymer used here was temperature-responsive, leading to the formation of dynamic higher order DNA–polymer structures in solution.

All of this work demonstrates the potential of DNA-polymer conjugates to provide access to highly functional materials. However, a significant barrier to greater uptake of the technology is the inefficiency with which DNA–polymer conjugates are produced. Where yields are reported in the literature they are typically below 20%, and conjugation with more hydrophobic polymers rarely exceeds 10% yield. This is problematic given the high cost of functional DNA as a starting material. Many reported syntheses also rely on the use of a solid support^8^,^9^,^10^,^18^,^19^,^20^, which usually implies the use of a DNA synthesizer, an expensive piece of equipment to which many polymer synthesis groups do not have access. It would be ideal therefore to find a method for creating DNA–polymer conjugates meeting the following criteria: i) Highly efficient; ii) Proceeds in a range of common organic solvents (to facilitate conjugation of more hydrophobic polymers); iii) Uses DNA starting materials that are commercially available and/or straightforward to produce; iv) Works in solution without the need for a solid support.

We previously reported that correct catalyst selection could make the copper-catalysed azide-alkyne cycloaddition (CuAAC) reaction a highly efficient chemistry for DNA–polymer conjugation in organic solvents^17^. We therefore set out to explore whether other macromolecular coupling strategies could be applied to DNA–polymer conjugation. In this work, the use of amide coupling chemistry, the thiol–ene reaction and the inverse electron demand Diels–Alder (DA_*inv*_) reaction were surveyed as potential routes to DNA–polymer conjugates. All of these chemistries have previously been used successfully for macromolecular coupling in solution in the absence of a solid support. For example, amide coupling has been successfully used for the synthesis of DNA–polymer conjugates in water, with yields of up to 90%^21^,^22^,^23^,^24^,^25^,^26^. We could find only one reported yield for the use of this reaction to produce conjugates in organic solution (DMSO in this case), which was tantalisingly high at 75%^27^. The use of the thiol Michael addition for synthesis of diblock copolymers of poly(*N*-isopropylacrylamide) (Poly(NIPAM))and PS was reported by Sumerlin *et al*. and the use of this reaction in polymer chemistry has been extensively reviewed^28^,^29^. Kataoka *et al*. reported DNA conjugates with poly(ethylene glycol) (PEG) in yields up to 89% in water, while Herrmann *et al*. synthesised DNA–PS conjugates with yields of around 10%^30^,^31^,^32^,^33^,^34^. Hansell *et al*. used the DA_*inv*_ reaction between tetrazine (Tz) and norbornene (Nb) to couple polymers synthesised *via* different routes with high efficiency under ambient conditions^35^. While these examples illustrate that these chemistries hold promise, to our knowledge no systematic study exists of their applicability to the synthesis of DNA–polymer conjugates.

Reversible Addition-Fragmentation Chain Transfer (RAFT) polymerisation was used for this work as it provides good control over molecular weight for a wide range of monomers^36^. Poly(NIPAM) was chosen as a model polymer as it is organic- and water-soluble but also exhibits temperature-responsive behaviour, a potentially useful characteristic for nanotechnology applications^37^.

## Results and Discussion

**Figure 1:**
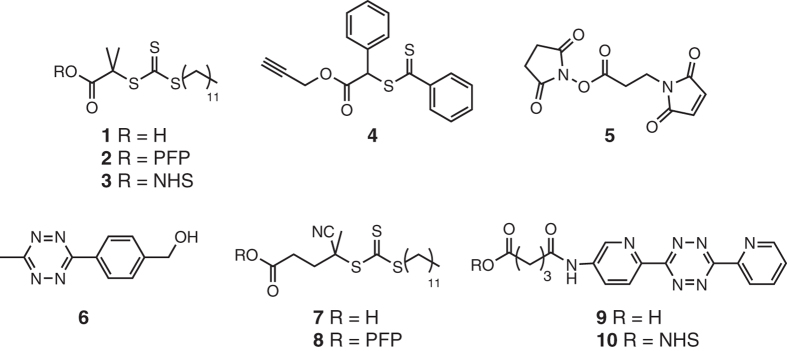
Structures of the small molecules used for this study.

The properties of the polymers used in these studies are given in Table 1. A summary of the coupling conditions tested is presented in Table 2. Structures of small molecules are given in Fig. 1.

### Amide Coupling

Since the thiocarbonyl thio groups present in RAFT chain transfer agents (CTAs) degrade in the presence of amines, it has always been difficult to incorporate this group into polymers synthesised using the RAFT technique^38^. By contrast, carboxylic acid groups are stable under RAFT polymerisation conditions and can be incorporated into the CTA. 2-(Dodecylthiocarbonothioylthio)-2-methylpropionic acid (DDMAT, **1**) is a widely used CTA, which has been shown to control the polymerisation of a large variety of monomers, and which contains a free carboxylic acid group in its structure (Fig. 1). It was envisaged that polymers produced using this CTA could be coupled to amine-functionalised DNA.

Poly(NIPAM) of varying molecular weights was thus synthesised using RAFT polymerisation, with **1** as the CTA. As outlined in Table 1, good control over molecular weight was achieved, with all polymers having a low dispersity (i.e. below 1.2). It was thought that the trithiocarbonate end group might interfere with the DNA coupling reaction, so, using a previously reported method^39^, this group was substituted with a hydrogen atom (Supplementary Figure S1), effectively removing all functionality from this end of the polymer. Successful end group removal was confirmed by comparing the SEC UV traces at 309 nm (characteristic of the trithiocarbonate group) before and after the reaction (Supplementary Figure S1). ^1^H NMR spectroscopic analysis in *d*_6_-DMSO confirmed that the carboxylic acid group survived the end group removal process (Supplementary Figure S2).

With the carboxylic acid-functionalised polymers in hand, coupling of amine-functionalised DNA (**s0**-**NH**_2_) was attempted. The DNA strand **s0**-**NH**_2_ was designed to have no secondary structure at room temperature and was 22 nucleotides in length (see Supplementary Figure S3 for sequence).

Since the primary aim of this work was to find an accessible way of conjugating polymers to DNA, it was decided to work at DNA concentrations not above 10 *μ*M. This meant that reactions could be carried out on a practical scale (i.e. 10 *μ*L or above) without using too much material – an important consideration given the high cost of functional DNA. Coupling to the carboxylic acid-functionalised poly(NIPAM) (**P1a**) synthesised above was attempted using a variety of solvents and coupling agents as summarised in Table 2.

The coupling agents and polymer (see Supplementary Figure S4 for structures) were added in a 10- to 100-fold excess relative to the amine group and the reaction left overnight to proceed. Each reaction mixture was then diluted with 5× glycerol loading buffer and water, and analysed by 15% native poly(acrylamide) gel electrophoresis (PAGE).

Tables S1–3 give the details of all the coupling reactions attempted. No product was observed by PAGE when EDCI, DCC, PyBOP or PyBroP were used as the coupling agent (Supplementary Figures S5-6). However, when HBTU or HATU were used (popular coupling reagents often recommended for ‘difficult’ coupling reactions)^40^ a slow-moving band attributed to the polymer–DNA conjugate was observed when DMF was used as the reaction solvent (Supplementary Figure S6). Analysis of the PAGE results using densitometry (Supplementary Figure S7) gave an approximate yield of the conjugate of 25%. The product bands were excised and MS analysis (ESI and MALDI-ToF) attempted, however it did not prove possible to obtain mass information about the extracted products. Instead, PAGE control experiments were performed to eliminate the possibility that these bands were caused by other species such as degradation products, dimers, or non-specific association of **s0** with poly(NIPAM) (Supplementary Figure S8). A large number of previous reports also assign a broad, slow-moving band to a conjugation product^8^,^10^,^11^,^12^,^13^,^14^,^15^,^16^,^17^,^21^,^22^,^23^,^24^,^25^,^26^,^27^,^30^,^31^,^32^,^33^,^34^.

Experiments were also carried out to determine whether the degree of polymerisation (DP) – and therefore the molecular weight – of the polymer used had any effect on the efficiency of the coupling reaction. To this end, the synthesis of conjugates containing poly(NIPAM) with DPs of 97 and 196 (**P1b** and **P1c**) was attempted using the conditions identified above, with HATU or HBTU as the coupling agent and DMF as the solvent. However, in no cases were the conjugates observed (Supplementary Figure S9). It also proved very difficult to repeat the synthesis of the lower DP conjugate, despite increasing the purity of the solvent and DNA. The best yield obtained in the repeat experiments was 5% (as assessed by PAGE densitometry), but only for the lower DP polymer **P1a**.

### Amide Coupling Using Pre-Activated Acids

Recently, pentafluorophenyl (PFP) esters have been used for the functionalisation of polymers produced by RAFT polymerisation^41^,^42^,^43^,^44^. The more established NHS esters have also been shown to be very useful in polymer functionalisation^45^. Previous work in our group has shown that pre-activated species such as these may be more effective for modification of **s0**-**NH**_2_ with small molecules than generating the activated ester *in situ*. It is hypothesised that this is because the formation of the activated ester results in the production of by-products, which later inhibit the attack of the amine and/or cause its degradation. It was therefore reasoned that conjugation of **s0**-**NH**_2_ to polymers containing a pre-activated acid group may be more efficient than the coupling agent-mediated process described above.

Two activated ester-functionalised CTAs (**2** and **3**) were thus synthesised. Both were used to polymerise NIPAM with good control over molecular weight and dispersity (see Table 1, **P2-3**). The presence of the PFP group was confirmed by ^19^F NMR spectroscopy by observing the appearance of broad signals at −152.9, −158.0 and −162.3 ppm attributable to the PFP group attached to the polymer.

By introducing an external standard with both fluorine and hydrogen NMR peaks (in this case trifluorotoluene), it was possible to estimate the degree of PFP functionalisation – around 80-95% (Supplementary Figure S10). The presence of the NHS group was confirmed by ^1^H NMR spectroscopy by comparing the integral of the signal at 2.85 ppm (attributable to the two CH_2_ groups on the succinimide group) to that of the signal at 3.30 ppm (due to the CH_2_ group adjacent to the trithiocarbonate group at the opposite end of the polymer). To prevent unwanted side reactions, the trithiocarbonate groups were removed from the termini of **P2** and **P3** using AIBN and LPO (Supplementary Figures S11–14)^46^.

To confirm the reactivity of the PFP group at the polymer chain end, a study using a small molecule amine was undertaken. **P3** was dissolved in one of three NMR solvents − *d*_7_-DMF, *d*_6_-DMSO or *d*_8_-THF – and a small excess of benzylamine was added. The reactions were left overnight at room temperature. ^1^H NMR analysis showed the appearance of a new peak around 5 ppm (the exact value was solvent-dependent) due to the CH_2_ group adjacent to the newly-formed amide group, and ^19^F NMR showed the complete absence of any PFP ester peaks, concomitant with the appearance of new peaks due to the pentafluorophenol formed during the reaction (Supplementary Figure S15). It was therefore concluded that the PFP end group was present and reactive under mild conditions towards primary amines.

Having confirmed that the activated esters remained intact following end group removal, coupling to **s0**-**NH**_2_ was then attempted under various reaction conditions (summarised in Table 2, detailed conditions in Supplementary Table S4). However, under none of these conditions was the expected product observed. It is clear that, under these conditions, neither of the activated esters used were reactive enough for the DNA conjugate to form. Instead, hydrolysis and/or degradation occurred before the attack of the amine. Given the low yields and irreproducible nature of the conjugation reactions, it was concluded that amide coupling was not effective for the production of DNA-polymer conjugates in organic solvents.

### Thiol–Ene Michael Addition

DNA–polymer conjugation was previously reported using the thiol–maleimide^32^ and thiol–acrylate^31^ Michael addition reactions, but only ever in an aqueous environment. Yields were good (around 90% in the case of the maleimide reaction) but required a relatively high DNA concentration of 0.1 mM, and the use of water as the reaction solvent precluded the conjugation of hydrophobic polymers. Given that polymer–polymer coupling in organic solvents has been achieved using thiol Michael addition chemistry, it was reasoned that an exploration of reaction conditions could yield some interesting results in the area of DNA–polymer conjugation. DNA strands functionalised with a methacrylamide, acrylamide or maleimide group were therefore targeted in the following work, and tested for their reaction with thiol-terminated polymers.

Thiol-terminated polymers were synthesised by stirring **P1a-c** with sodium borohydride and purified by dialysis. Successful removal of the trithiocarbonate end group was confirmed by SEC (Supplementary Figure S16). Some disulfide coupling was observed, but the presence of the free thiol was confirmed using Ellman’s assay (see Table 1 for a summary of the thiol contents)^47^,^48^. DNA functionalised with a methacrylamide group (Supplementary Figure S17) was commercially available, so the s0 sequence was purchased with this modification at the 5′ end (**s0**-**MAAm**). Conjugation to thiol-terminated **P4a-c** was then attempted. However, despite testing many different catalyst and solvent combinations (Table 2 and Tables S5-8), negligible product was formed (Figs S18-20).

It was thought that formation of the thiol group should be attempted *in situ*, so poly(NIPAM) containing a terminal dithioester group (**P5**, synthesised using alkyne-containing CTA **4**) was mixed with **s0**-**MAAm** in the presence of hexylamine and TEA, which should have cleaved the dithioester to release a thiol – these reagents also served to catalyse the thiol Michael addition reaction itself. Again, several solvents were tested as outlined in Table 2. However, no product was observed.

Having achieved only very low yields using the methacrylamide-functionalised DNA, the use of the more reactive acrylamide group was explored. Acrylamide-functionalised DNA was not commercially available, so it was synthesised from **s0**-**NH**_2_ DNA and acrylic acid using EDCI and HOBt as coupling agents. Purification of the reaction mixture by HPLC (Supplementary Figure S21) afforded the product, which was analysed by LC-MS and had the expected mass. Identical polymer couplings were attempted, but no product was observed in any case. It was decided that a different type of ene compound should be used, so maleimide was investigated as a potentially more active alternative to (meth)acrylamide.

Maleimide-functionalised DNA (**s0**-**Mal**) was not commercially available, so the desired product was synthesised using a bifunctional adapter, **5**. The product was isolated via HPLC and LC-MS confirmed the expected mass (Supplementary Figure S22). Polymer conjugation reactions were first attempted under aqueous conditions (Supplementary Table S9) and broad, low-mobility bands were observed (Supplementary Figure S23), with estimated yields of around 50%.

Having successfully formed the DNA–polymer conjugate in an aqueous environment, the use of organic solvent systems was explored. Five different organic solvents were tested, both with and without an auxiliary base, as summarised in Table 2 (detailed conditions in Supplementary Table S10). No product was observed for any of the solvents trialled (Supplementary Figure S24) and HPLC analysis confirmed that degradation of the maleimide group was taking place (Supplementary Figure S25).

Having attempted DNA–polymer conjugation using methacrylamide-, acrylamide- and maleimide-functionalised DNA in a number of organic solvents and observed only very slight evidence of the formation of a product, it was concluded that the thiol Michael addition reaction was not going to provide a successful route to the desired conjugates.

### Tetrazine–Norbornene Coupling

Recent work in our group has revealed that the DA_*inv*_ reaction between Tz and Nb is efficient for the conjugation of macromolecules in solution^35^. Nb-functionalised DNA (**s0**-**Nb**) was synthesised using an acid-containing Nb and **s0**-**NH**_2_ under standard EDCI/HOBt coupling conditions. After one hour, HPLC analysis indicated the formation of two products in nearly quantitative yield (Supplementary Figure S26-27), which were both found to have the same mass of 6 996 Da, which was the expected mass of **s0**-**Nb**; it was therefore concluded that the two products corresponded to the *endo*- and *exo*-Nb adducts (the Nb acid starting material was a mixture of the two isomers).

The reactivity of the Nb group on the DNA was tested by mixing each of the **s0**-**Nb** isomers with an alcohol-functionalised Tz (**6**) under conditions analogous to those to be used for the DNA–polymer conjugation. Analysis of the DNA by HPLC before and after addition of **6** revealed a clear peak shift for both isomers (Supplementary Figure S28), indicating that both retained an intact Nb group and that this group was reactive towards Tz. UV-vis spectroscopy also confirmed that the reaction had taken place (Supplementary Figure S29).

The Tz group is not stable towards radical polymerisation conditions as it reacts with monomers bearing vinyl functionality^49^. Incorporation of this group into an appropriate CTA was therefore not a viable option for the production of Tz-functionalised polymers by RAFT polymerisation. The alcohol-containing Tz, **6**, was therefore used in the post-polymerisation modification of PFP-terminated poly(NIPAM), as above. Functionalisation of **P3** was found not to proceed, possibly because the two methyl groups *α* to the PFP ester were hindering the attack of the alcohol – PFP-methacrylate has been observed to react with alcohols much more slowly (if at all) than the acrylate analogue^50^. An alternative PFP-containing CTA, **8**^51^, was therefore synthesised wherein the PFP group was held at the end of an ethyl linker, minimising steric blocking. Using pentafluorophenyl trifluoroacetate as the source of the PFP group, the yield was dramatically improved over the literature preparation (80% vs. 20%).

Next, the CTA **8** was used to control the RAFT polymerisation of NIPAM. Good control was achieved over molecular weight and dispersity (**P6**, see Table 1). ^1^H and ^19^F NMR analyses confirmed the presence of the end groups in the product (Figs S30–31).

**Figure 2:**
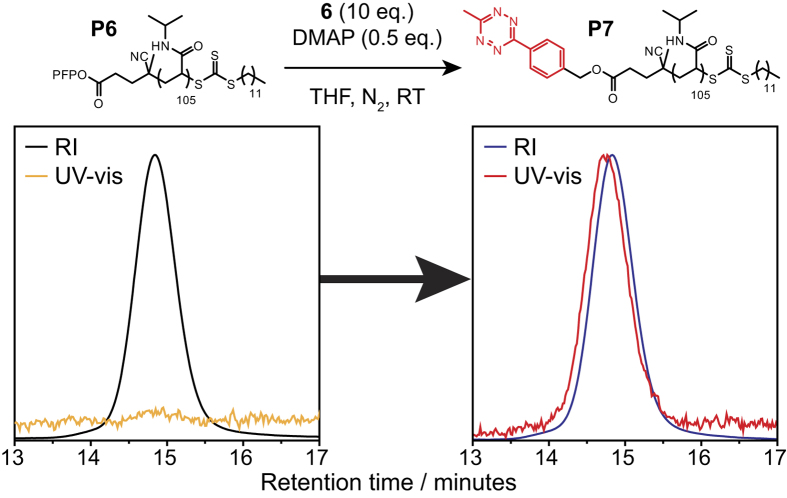
DMF SEC chromatograms showing the incorporation of the Tz group at the chain end of poly(NIPAM). There is the clear appearance of a peak in the UV-vis trace collected at 540 nm (characteristic of the Tz group).

Alcohol **6** was then mixed with **P6** and DMAP and stirred for twenty-four hours in anhydrous THF under an atmosphere of nitrogen. The polymer was then separated from the excess alcohol by preparatory SEC in DMF. Removal of the solvent under high vacuum yielded a pink solid (**P7**) which was studied by UV-vis spectroscopy and found to have the same peaks as the starting material, suggesting that the Tz group had been incorporated successfully (Supplementary Figure S32).^1^H NMR spectroscopy also showed the presence of new peaks due to the Tz group and the CH_2_ group adjacent to the newly-formed ester (Supplementary Figure S33), confirming the incorporation of the Tz group in approximately 50% yield. The polymer was also analyzed by DMF SEC with an in-line UV-vis detector set to 540 nm (the wavelength of one of the characteristic peaks of the Tz group). The results (Fig. 2) showed both that the molecular weight and dispersity of the polymer remained unchanged, and that the Tz group had been successfully incorporated at the polymer chain end, by the appearance of a peak at 540 nm.

Further confirmation was provided by running THF SEC analysis using a photodiode array (PDA) detector. This collected a full UV-vis spectrum for every retention time point. The full Tz peak at 540 nm was observed to elute at the same time as the main polymer peak (Supplementary Figure S34). Importantly, both ^1^H NMR spectroscopy and SEC indicated that no small molecule Tz was present in the sample.

**Figure 3:**
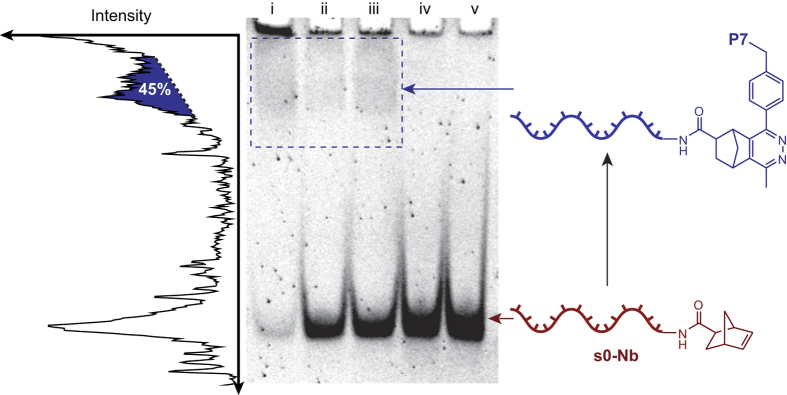
15% native PAGE analysis of the conjugation of **P7** to **s0-Nb**. The DNA-polymer conjugate was visible as a slow-migrating band (blue box) when water, DMF or DMAc were used as the reaction solvent (lanes i-iii), but not with NMP (lane iv) or DMSO (lane v). Left: Densitometry plot of lane i showing the band assigned to the DNA-polymer conjugate - the yield was calculated by comparing the areas under the conjugate and free **s0-Nb** peaks.

With the Tz-functionalised polymer (**P7**) in hand, coupling to **s0**-**Nb** was attempted under the conditions summarised in Table 2. The reaction mixtures were analysed by 15% native PAGE (Fig. 3), which showed that the conjugate was formed in moderate yield in water by the clear appearance of a low-mobility band attributed to the product. When DMF or DMAc was used as the reaction solvent, yields were below 10%, and in NMP and DMSO no reaction was observed. It should be noted at this point that in most cases PAGE analysis showed the retention of some DNA-containing material in the wells. Control experiments confirmed that this was due to the high concentration of free polymer in the loaded samples and did not effect the yield estimates – the higher concentration images are presented here as the product bands are easier to discern.

In an effort to improve the yields, the positions of the Tz and Nb groups were switched. In order to obtain Tz-functionalised DNA, it was first necessary to synthesise an adapter containing the Tz group. This was obtained by activating the Tz-acid, **9**, with *N*-hydroxysuccinimide (NHS) to give Tz-NHS, **10**. ^1^H (Supplementary Figure S35) and ^13^C NMR spectroscopy both showed the expected peaks, including the four protons from the NHS group.

Next, **10** was reacted with **s0**-**NH**_2_ in 1:1 DMF/PBS solution at 40 °C, with a DNA concentration of 100 *μ*M and a one thousand-fold excess of the small molecule. Purification of the reaction mixture yielded a peak which exhibited the characteristic Tz UV-vis absorbance at around 330 nm (Supplementary Figure S36), and had the expected mass (Supplementary Figure S37).

To confirm the reactivity of **s0**-**Tz**, a small molecule test reaction was carried out in both water and DMF. Commercially available 5-norbornene-2-*exo*,3-*exo*-dimethanol was mixed with **s0**-**Tz** at a concentration of 50 *μ*M and the reaction followed by HPLC. A clear peak shift was observed in both solvents (Supplementary Figure S38), confirming that the Tz retained its reactivity once conjugated to the DNA strand. The yield of the reaction was estimated by comparison of the areas under the peaks due to the Tz-Nb coupling product and unreacted DNA and found to be approximately 70% and 40% in water and DMF respectively. The difference in yield can possibly be attributed to degradation of the Tz group by free amines present in the DMF used.

**Figure 4:**
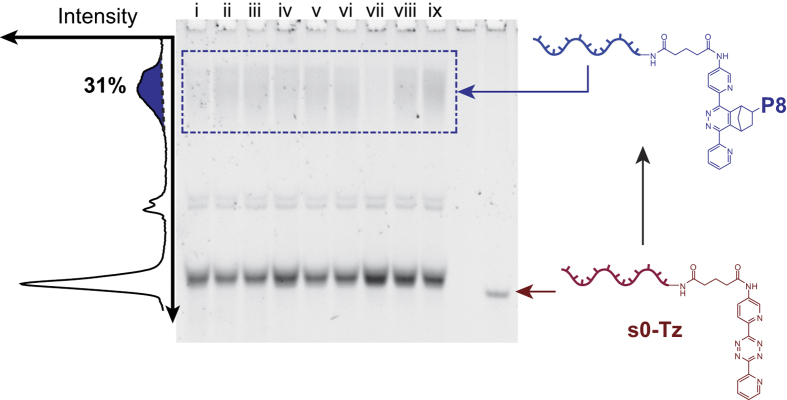
15% native PAGE analysis showing the formation of a DNA-polymer conjugate from **P8** and **s0-Tz** using the Tz-Nb DA_*inv*_ reaction. Left: Densitometry plot of lane ix showing the band assigned to the DNA-polymer conjugate - the yield was calculated by comparing the areas under the conjugate and free **s0-Tz** peaks. The solvents used were DMF (lanes i-iii), DMAc (lanes iv-vi) and NMP (lanes vii-ix), with either 1 (lanes i, iv and vii), 10 (lanes ii, v and viii) or 100 (lanes iii, vi and ix) equivalents of the polymer.

Next, DNA–polymer conjugation was attempted using poly(NIPAM) end-capped with a Nb group (**P8**)^52^. After reacting with 10 *μ*M **s0**-**Tz** DNA overnight at room temperature in DMF, DMAc or NMP (see Table 2), 15% native PAGE analysis revealed that the conjugate had been formed in up to 50% yield (by densitometry) – see Fig. 4. It should be noted that a significant shift in the migration distance of the starting material band was observed in these gels. This was thought to be due to degradation of the Tz group under the reaction conditions, and inspection of the HPLC analysis in Supplementary Figure S38 confirmed that this was likely the case: as well as the product peak a large number of smaller peaks appeared, which were attributed to degradation products. The significant difference in yield compared to the **s0-Nb**/**P7** reaction was attributed to the different structures of the Tz groups used, in line with recent reports, which show that in general electron-donating groups (such as the methyl group attached at the 6 position to the Tz in **P7**) increase the energy of the LUMO, making the reaction less favorable, while electron-withdrawing groups (such as the pyridyl groups of **s0-Tz**) lower the LUMO’s energy, increasing the favorability of the reaction^53^,^54^. The Tz-Nb method represented by far the most efficient DNA–polymer conjugation chemistry tested and is a novel, catalyst-free approach for the production of DNA–polymer conjugates in good yield in organic solution. Future studies will focus on elucidating the relationship between Tz structure and the efficiency of the reaction in different solvent systems in order to optimize yields.

## Conclusions

This work has demonstrated that the conjugation of polymers to DNA strands in organic solvents is not straightforward. At moderate DNA concentrations and in free solution both the thiol–ene reaction and the amide coupling route failed to yield significant amounts of the desired conjugate when organic solvents were used, despite their efficacy in water. While it was not possible to produce conjugates using these reactions a number of novel DNA and polymer modifications were developed which it is hoped will be of use in other areas of materials science, including acrylamide-functionalised DNA. In contrast, it was shown that the DA_*inv*_ reaction between a norbornene and a tetrazine can lead to the production of DNA–polymer conjugates in organic solution, at practical (i.e. reasonably low) DNA concentrations and without the need for a solid support. With further development of the precise structures of the norbornene and tetrazine derivatives used this technique has the potential to become a mild and high-yielding route to DNA–polymer conjugates.

## Methods

For materials and methods information and comprehensive experimental details please see the Supporting Information. Syntheses of key compounds are presented below.

### Synthesis of PFP-DDMAT, 2

PFP-DDMAT, **2**, was synthesised as follows. **1** (0.500 g, 1.37 mmol) was added to an oven-dried schlenk flask, which was then evacuated and refilled with nitrogen three times. Anhydrous DMF (7.5 mL) was added via syringe and the flask cooled to 0 °C with an ice bath. DIPEA (354 *μ*L, 2.74 mmol) was then added via syringe, followed by dropwise addition of pentafluorophenyl trifluoroacetate (283 *μ*L, 1.65 mmol). After one hour stirring at 0 °C, the flask was opened to the air and diethyl ether (30 mL) was added, followed by a 1 M solution of HCl (30 mL). The organic layer was collected and washed with water (2 × 30 mL) and brine (30 mL). The solvent was removed *in vacuo* to give a yellow oily residue, which was then purified by silica gel column chromatography, eluting with a mixture of ethyl acetate and pet. ether 40-60 (gradient from 5-10% ethyl acetate). The fractions containing the product (R_*f*_ = 0.81) were combined and the solvent removed *in vacuo* to yield CTA **2** as a yellow oil (0.686 g, 94%). ^1^H NMR (400 MHz, CDCl_3_) *δ* 3.31 (t, *J* = 7 Hz, 2H, SC*H*_2_), 1.86 (s, 6H, C(C*H*_3_)_2_), 1.69 (quint, *J* = 7 Hz, 2H, SCH_2_C*H*_2_), 1.40 (m, 2H, C*H*_2_CH_3_), 1.26 (br s, 16H, SCH_2_CH_2_(C*H*_2_)_8_), 0.88 (t, *J* = 7 Hz, 3H, CH_2_C*H*_3_) ppm. ^13^C NMR (150 MHz, CDCl_3_) *δ* 219.9 (*C* = S), 169.6 (*C* = O), 142.1 (t), 140.4 (t), 138.7 (t), 137.0 (t) (PFP *C*s), 55.4 (*C*(CH_3_)_2_), 37.2 (S*C*H_2_), 31.9, 29.6, 29.5, 29.4, 29.3, 29.1, 29.0, 28.9, 27.8, 25.4 (C(*C*H_3_)_2_), 22.7 (*C*H_2_CH_3_), 14.1 (S(CH_2_)_11_*C*H_3_) ppm. ^19^F NMR (375 MHz, CDCl_3_) −151.5 (d, 2F, ortho *F*), −157.7 (t, 2F, para *F*), −162.3 (t, 2F, meta *F*) ppm. IR (*ν*_*max*_/cm^−1^): 2925, 2854, 1779, 1517, 1079, 992, 815. ESI HR MS calcd. for C_23_H_31_F_5_O_2_S_3_ [M + H]^+^ 531.1486 Da; observed 531.1480 Da.

### Synthesis of NHS-DDMAT, 3

NHS-DDMAT, **3**, was synthesised as follows^45^. **1** (0.500 g, 1.37 mmol), NHS (0.158 g, 1.37 mmol) and DCC (0.283 g, 1.37 mmol) were dissolved in dichloromethane and the mixture stirred for 48 hours. The cloudy mixture was filtered through a 0.45 *μ*m PTFE syringe filter and the retentate washed with CH_2_Cl_2_ (5 mL). This filtration process was then repeated. The solvent was removed *in vacuo* and the residue dissolved in a small amount of ethyl acetate. The solution was purified by silica gel column chromatography, eluting with a mixture of ethyl acetate and pet. ether 40–60 (1:1). The product fractions (R_*f*_ = 0.53) were collected and the solvent removed *in vacuo* to afford **3** as a yellow solid (0.502 g, 79%). ^1^H NMR (400 MHz, CDCl_3_) *δ* 3.30 (t, *J* = 7 Hz, 2H, SC*H*_2_), 2.81 (br s, 4H, C*H*_2_(C = O)N), 1.87 (s, 6H, C(C*H*_3_)_2_), 1.68 (quint, *J* = 7 Hz, 2H, SCH_2_C*H*_2_), 1.38 (m, 2H, C*H*_2_CH_3_), 1.25 (br s, 16H, SCH_2_CH_2_(C*H*_2_)_8_), 0.88 (t, *J* = 7 Hz, 3H, CH_2_C*H*_3_) ppm. ^13^C NMR (150 MHz, CDCl_3_) *δ* 218.8 (*C* = S), 169.1 (*C* = OO), 168.7 (NHS *C* = O), 54.3 (*C*(CH_3_)_2_), 37.2 (S*C*H_2_), 31.9 (SCH_2_*C*H_2_), 29.6, 29.5, 29.4, 29.3, 29.1, 29.0, 27.8, 25.4 (NHS *C*H_2_), 25.3 (C(*C*H_3_)_2_), 22.7 (*C*H_2_CH_3_), 14.1 (S(CH_2_)_11_*C*H_3_) ppm. IR (*ν*_*max*_/cm^−1^): 2917, 2848, 1777, 1736, 1203, 1074, 811. ESI HR MS calcd. for C_21_H_35_NO_4_S_3_ [M + Na]^+^ 484.1626 Da; observed 484.1619 Da.

### Synthesis of P2 and P3 using CTAs 2 and 3

Polymerisation of NIPAM with **3** was conducted as follows. NHS-DDMAT, **3**, (0.041 g, 0.09 mmol), NIPAM (1.000 g, 8.84 mmol) and AIBN (0.002 g, 0.01 mmol) were dissolved in 1,4-dioxane (1.5 mL) and transferred to an oven-dried ampoule. The mixture was subjected to three freeze-pump-thaw cycles and sealed under an atmosphere of nitrogen. It was then placed in an oil bath preheated to 65 °C. After 2 hours the ampoule was removed and the reaction quenched by opening it to the air and cooling with liquid nitrogen. The solution was poured into pet. ether 40-60 (80 mL) cooled in an ice bath and the precipitant collected by filtration. The product was then dissolved in THF (1 mL) and the process repeated 5 more times. Finally, the isolated solid was dissolved in THF (1 mL) and precipitated into diethyl ether (80 mL) cooled in an ice bath. The product was isolated by filtration, dried *in vacuo* and isolated as a yellow powder (0.335 g, 36%) and analysed by DMF SEC using PMMA calibration standards (M_*n*_ 9 610 Da, Đ 1.10). ^1^H NMR (400 MHz, CDCl_3_) *δ* 7.36–5.55 (br m, PNIPAM N*H*), 4.00 (br s, PNIPAM C*H*(CH_3_)_2_), 3.33 (br m, 2H, SC*H*_2_), 2.86 (br s, 4H, C*H*_2_(C = O)N), 2.64–0.80 (br m, PNIPAM backbone *H*), 0.88 (t, J = 7 Hz, 3H, S(CH_2_)11C*H*_3_) ppm.

PFP-DDMAT, **2**, was also used to polymerise NIPAM using an identical procedure.

### Synthesis of s0-AAm

Acrylamide-functionalised DNA (**s0**-**AAm**) was synthesised from amine-functionalised DNA (**s0**-**NH**_2_) as follows. Acrylic acid (16.7 *μ*L, 600 mM in DMF, 10 *μ*mol), EDCI (16.7 *μ*L, 600 mM in DMF, 10 *μ*mol), HOBt (16.7 *μ*L, 600 mM in DMF, 10 *μ*mol) and DIPEA (1.7 *μ*L, 10 *μ*mol) were mixed and incubated at room temperature for thirty minutes. Phosphate buffer pH 8.0 (47.0 *μ*L) and **s0**-**NH**_2_ (3.2 *μ*L, 3.17 mM in water, 10 nmol) were added and the mixture left at room temperature for 24 hours. The excess small molecules and DMF were removed by extraction with dichloromethane (3 × 200 *μ*L). The aqueous layer was isolated and topped up to a final volume of 100 *μ*L with water. The product was isolated by HPLC, with a yield of 20% as quantified by UV-vis spectroscopy using the known extinction coefficient of the starting material DNA at 260 nm.

### Synthesis of s0-Mal using the bifunctional adapter, 5

**s0**-**NH**_2_ (1000 *μ*L, 200 *μ*M in water, 200 nmol), **5** (53.6 mg, 200 mmol) and DIPEA (35 *μ*L, 200 mmol) were mixed in DMF (1000 *μ*L) and the reaction shaken overnight at 40 °C. The excess small molecules were then removed using a NAP-10 Sephadex column and the collected solution concentrated *in vacuo* and purified by HPLC. The product was isolated as a single fraction, with an isolated yield of 63% as quantified by UV-vis spectroscopy using the known extinction coefficient of the starting material DNA at 260 nm.

### Synthesis of s0-Nb

EDCI (100 *μ*L, 300 mM in DMF) was mixed with HOBt (100 *μ*L, 300 mM in DMF), 5-norbornene-2-carboxylic acid (100 *μ*L, 300 mM in DMF) and PBS (150 *μ*L) and thoroughly mixed. 75 *μ*L of this solution was mixed with **s0**-**NH**_2_ (25 *μ*L, 200 *μ*M in water) and DIPEA (0.87 *μ*L). After one hour shaking the flask at room temperature the mixture was purified by HPLC and the product isolated as two separate peaks. Both were analysed by MALDI-ToF MS. Expected mass 6 996.3 Da; observed 6 995.8 Da.

### Perfluorophenyl 4-cyano-4-(dodecylthiocarbonothioylthio)-pentanoate, 8

Perfluorophenyl 4-cyano-4-(dodecylthiocarbonothioylthio)pentanoate (**8**) was synthesised as follows^51^. **7** (0.5 g, 1.24 mmol) was added to an oven-dried flask under nitrogen. Anhydrous DMF (9 mL) was added followed by DIPEA (431 *μ*L, 2.48 mmol). The mixture was cooled using an ice bath and pentafluorophenyl trifluoroacetate (255 *μ*L, 1.49 mmol) was added dropwise with vigorous stirring. The reaction was allowed to proceed for one hour, then diethyl ether (40 mL) was added followed by 1 M HCl (40 mL). The organic layer was separated and washed with water (2 × 40 mL) and brine (40 mL). The solvent was then removed *in vacuo* and the residue purified by silica gel column chromatography, eluting with a mixture of hexane and diethyl ether (10:1). The product (R_*f*_ 0.21) was isolated as an orange viscous liquid (0.563 g, 80%). ^1^H NMR (300 MHz, CDCl_3_) *δ* 3.34 (t, *J* = 7 Hz, 2H, SC*H*_2_), 3.01 (m, 2H, C*H*_2_CO_2_PFP), 2.60 (m, 2H, C*H*_2_CH_2_CO_2_PFP), 1.93 (s, 3H, SCC*H*_3_), 1.70 (quint, *J* = 7 Hz, 2H, SCH_2_C*H*_2_), 1.40 (m, 2H, SCH_2_CH_2_C*H*_2_), 1.26 (br s, 16H, S(CH_2_)_3_(C*H*_2_)8CH_3_), 0.88 (t, *J* = 7 Hz, 3H, S(CH_2_)11C*H*_3_) ppm. IR (*ν*_*max*_/cm^−1^) 2918, 2850, 1797, 1517, 1094, 990, 803. (These values compare well with the literature values above, but are given because a significantly different method was used for the synthesis).

### 2,5-dioxopyrrolidin-1-yl 5-*oxo*-5-(6-(6-(pyridin-2-yl)-1,2,4,5-tetrazin-3-yl)pyridin-3-ylamino)pentanoate, 10

The tetrazine DNA adaptor was synthesised as follows. Tetrazine **9** (0.100 g, 0.27 mmol) and NHS (0.032 g, 0.27 mmol) were dissolved in DMF (4 mL) and the solution bubbled with nitrogen for 30 minutes. The mixture was then cooled using an ice bath and DCC (0.057 g, 0.27 mmol) dissolved in DMF (1 mL) was added via syringe. After 30 minutes stirring the ice bath was removed and the reaction stirred under nitrogen for 16 hours. The reaction mixture was adsorbed onto silica which had been previously treated with EtSiCl_3_. EtSiCl_3_-treated silica gel column chromatography was then performed, eluting first with DMF, then acetone. The pure fractions were collected and combined, then dried *in vacuo* to afford the product **10** as a deep red powder (0.048 g, 38%). ^1^H NMR (400 MHz, *d*_6_-DMSO) *δ* 10.67 (brs, 1H, N*H*C = O), 9.10 (d, *J* = 2 Hz, 1H, Tz *H*1), 8.97 (d, *J* = 4 Hz, 1H, Tz *H*7), 8.66 (d, *J* = 9 Hz, 1H, Tz *H*4), 8.63 (d, *J* = 8 Hz, 1H, Tz *H*3), 8.47 (dd, *J* = 2, 9 Hz, 1H Tz *H*2), 8.19 (td, *J* = 1, 4 Hz, 1H, Tz *H*6), 7.76 (dd, *J* = 5, 7 Hz, 1H, Tz *H*5), 2.87 (m, 6H, C*H*_2_(C = O)N and C*H*_2_(C = O)NH), 2.63 (t, *J* = 7 Hz, 2H, C*H*_2_(C = O)OSu), 2.03 (quint, *J* = 7 Hz, 2H, C*H*_2_CH_2_(C = O)NH) ppm. ^13^C NMR (125 MHz, *d*_6_-DMSO) *δ* 172.5 (*C* = ONH), 170.7 (NHS *C* = O), 169.3 (*C* = OO), 163.5 (Tz N-*C* = N), 163.2 (Tz N-*C* = N), 151.1 (Tz *C*7), 150.7 (Tz C-*C* = N), 144.3 (Tz C-*C* = N), 141.8 (Tz *C*1), 138.9 (Tz *C*-NHC = O), 138.3 (Tz *C*5), 127.0, 126.7, 125.4, 124.7 (Tz *C*2/3/4/6), 35.1 (*C*H_2_C = ONH), 30.0 (*C*H_2_C = OO), 25.9 (NHS *C*H_2_), 20.2 (*C*H_2_CH_2_C = O) ppm. IR (*ν*_*max*_/cm^−1^): 2895, 1732, 1714, 1543, 1392, 1061. ESI HR MS calcd. for C_21_H_18_N_8_O_5_ [M + Na]^+^ 485.1298; observed 485.1293.

### Synthesis of s0-Tz

300 mM solutions of EDCI, HOBt and **10** were prepared in DMF and then mixed in equal proportions. 100 *μ*L of **s0**-**NH**_2_ (200 *μ*M in water) were added to a 1 mL centrifuge tube and the solvent removed *in vacuo*. 100 *μ*L of the EDCI/HOBt/**10** mixture were added, followed by 100 *μ*L of potassium phosphate buffer (100 mM, pH 8.0). The solution was vortexed to mix and then heated at 40 °C for four hours, after which time small molecules were removed by passing the solution through a NAP-5 sephadex column, eluting with water. The sample was concentrated *in vacuo* and then purified by HPLC. The product was isolated as a single peak (6%) and analysed by LC-MS. Expected mass 7 223.9 Da; observed 7 223.2 Da.

## Additional Information

**How to cite this article**: Wilks, T. R. and O’Reilly, R. K. Efficient DNA–Polymer Coupling in Organic Solvents: A Survey of Amide Coupling, Thiol–Ene and Tetrazine–Norbornene Chemistries Applied to Conjugation of Poly(*N*-Isopropylacrylamide). *Sci. Rep.* **6**, 39192; doi: 10.1038/srep39192 (2016).

**Publisher's note:** Springer Nature remains neutral with regard to jurisdictional claims in published maps and institutional affiliations.

## Supplementary Material

Supplementary Information

## Figures and Tables

**Table 1:** Poly(NIPAM) samples produced by RAFT polymerisation for this study.

Polymer	CTA	DP^*NMR*^	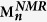 /kDa	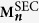 /kDa	Đ	Other Information
**P1a**	**1**	50	6.0	5.3	1.14	—
**P1b**	**1**	97	11.3	10.4	1.14	—
**P1c**	**1**	196	22.5	20.0	1.18	—
**P2**	**2**	98	11.1	9.6	1.10	>99% PFP incorporation
**P3**	**3**	78	8.8	7.8	1.10	80–95% NHS incorporation
**P4a**	**1**	50	6.0	5.7	1.17	47% free thiol
**P4b**	**1**	97	11.3	11.7	1.23	37% free thiol
**P4c**	**1**	196	22.5	21.4	1.30	27% free thiol
**P5**	**4**	61	7.3	6.9	1.16	—
**P6**	**9**	105	12.5	11.5	1.05	98% PFP incorporation
**P7**	**9**	105	12.5	13.1	1.04	53% Tz incorporation
**P8**	**—**	145	16.4	—	1.12	>99% Nb incorporation

DMF was used as the SEC eluent in all cases, with poly(methyl methacrylate) standards. DP = Degree of polymerisation.

**Table 2:** Summary of the coupling chemistries, reagents, catalysts and solvents used for the attempted synthesis of DNA–Poly(NIPAM) conjugates.

DNA-X + Poly(NIPAM)-Y → DNA-Poly(NIPAM)					
X =	Y =	Solvents	Coupling Agent(s)	Catalyst(s)	Product Observed?
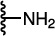	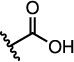	DMF, DMSO, MeCN, NMP, THF	EDCI, DCC, PyBOP, PyBroP	HOBt	No
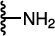	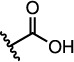	DMF, DMSO, MeCN, NMP, THF	HBTU, HATU	—	Yes*
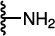	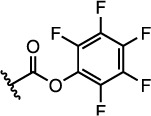	DMAc, DMF, NMP	—	—	No
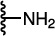	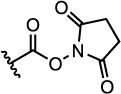	DMAc, DMF, NMP	—	—	No
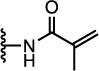	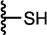	DMF, NMP	—	TCEP, DMPP	No
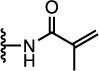	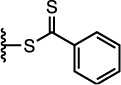	DMF, DMSO	Hexylamine	TEA	No
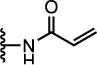	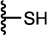	DMF, NMP	—	TCEP, DMPP	No
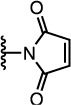	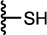	DMF, DMSO, MeCN, NMP, THF	DIEA	—	No
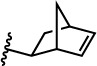	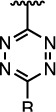	DMAc, DMF, DMSO, NMP	—	—	**Yes**
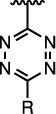	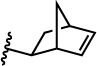	DMAc, DMF, NMP	—	—	**Yes**

^*^Results were not reproducible.
