# Supplementary material for: Efficient DNA–Polymer Coupling in Organic Solvents: A Survey of Amide Coupling, Thiol-Ene and Tetrazine–Norbornene Chemistries Applied to Conjugation of Poly(N-Isopropylacrylamide)
Source: Sci Rep. 2016 Dec 16;6:39192. doi: 10.1038/srep39192 (PMC5159856; doi:10.1038/srep39192)
Supplement: Supplementary Information [file srep39192-s1.pdf]

Supporting Information

Efficient DNA-Polymer Coupling in Organic Solvents: A Survey of Amide Coupling, Thiol–Ene and Tetrazine–Norbornene Chemistries Applied to Conjugation of Poly(*N*-Isopropylacrylamide)

Thomas R. Wilks and Rachel K. O'Reilly

Contents

1 Materials & Methods 2
1.1 Materials 2
1.2 Size exclusion chromatography (SEC) 3
1.3 NMR spectroscopy 3
1.4 IR spectroscopy 3
1.5 UV-vis spectroscopy 3
1.6 Mass spectrometry 3
1.7 Electrophoresis 3
1.8 HPLC 4
2 Experimental 4
2.1 Synthesis of poly(NIPAM) using DDMAT 4
2.2 Removal of the RAFT end group using EPHP 4
2.3 DNA–polymer conjugation using amide coupling reagents 5
2.4 Synthesis of PFP–DDMAT, 2 5
2.5 Synthesis of NHS–DDMAT, 3 5
2.6 Synthesis of poly(NIPAM) using NHS– and PFP–DDMAT 6
2.7 Removal of the trithiocarbonate group using AIBN and LPO 6
2.8 Attempted conjugation of P2 and P3 to s0–NH_2 6
2.9 Polymer end group removal using $NaBH_4$ 7
2.10 Ellman's assay 7
2.11 Conjugation of P4a-c to s0–MAAm without catalyst 7
2.12 Conjugation of poly(NIPAM)–SH to s0–MAAm with TCEP catalyst 7
2.13 *In situ* aminolysis of P5 in the presence of s0–MAAm 7
2.14 Conjugation of P4a-c to s0–MAAm with DMPP catalyst 7
2.15 Synthesis of s0–AAm 8

2.16 Conjugation of P4a-c to s0–AAm 8
2.17 Synthesis of s0–Mal using the bifunctional adapter, 5 8
2.18 Conjugation of P4a-c to s0–Mal 8
2.19 Conjugation of P4a-c to s0–Mal in organic solvents 8
2.20 RAFT polymerisation using CTA 4 8
2.21 Synthesis of s0–Nb 9
2.22 Reaction of s0–Nb with 6 9
2.23 Attempted modification of PFP-containing P2 with 6 9
2.24 Perfluorophenyl 4-cyano-4-(dodecylthiocarbonothioylthio)-pentanoate, 8 9
2.25 RAFT polymerisation of NIPAM using CTA 8 10
2.26 Modification of P6 with 6 10
2.27 Conjugation of s0–Nb to P7 10
2.28 2,5-dioxopyrrolidin-1-yl 5-oxo-5-(6-(6-(pyridin-2-yl)-1,2,4,5-tetrazin-3-yl)pyridin-3-ylamino)pentanoate, 10 11
2.29 Synthesis of s0–Tz 11
2.30 Conjugation of s0–Tz to P8 12
3 References 40

1 Materials & Methods

1.1 Materials

2-(Dodecylthiocarbonothioylthio)-2-methylpropionic acid (DDMAT, **1**) was synthesised using a previously published procedure and recrystallised from acetone/water.[1] *N*-isopropylacrylamide (NIPAM) was recrystallised from hexane and stored at 4°C. 2,2′-Azobis(2-methylpropionitrile) (AIBN) was purchased from Wako Pure Chemical Industries and recrystallised twice from methanol and stored in the dark at 4°C. Styrene was passed through a neutral alumina column prior to use to remove the radical inhibitor. 18 MΩ water was obtained using a MilliQ™ Simplicity system. Dialysis membranes were purchased from Spectra/Por® and soaked in 18 MΩ water before use. The DNA strands s0–NH_2 and s0′ were purchased from Integrated DNA Technologies, Inc. and re-suspended in 18 MΩ water to a concentration of 200 µM before use. Syringe filters were purchased from Gilson Scientific Ltd. Silica gel for column chromatography and all NMR solvents were purchased from Apollo Scientific Ltd. MOPS buffer consisted of 100 mM 3-(*N*-morpholino)propanesulfonic acid adjusted to the desired pH with 1 M HCl or NaOH. PBS consisted of 100 mM potassium phosphate – the desired pH was achieved by mixing appropriate amounts of the monobasic and dibasic versions (both at 100 mM). All other chemicals were purchased from Sigma-Aldrich Corporation and used as received. 3-Maleimidopropionic acid *N*-hydroxysuccinimide ester (**5**) was purchased from Alfa Aesar and used as received. NAP-5 and -10 sephadex columns were purchased from GE Healthcare. *S*-Propargyloxycarbonylphenylmethyl dithiobenzoate (**4**) was synthesised according to a previously published procedure. 5-oxo-5-(6-(6-(pyridin-2-yl)-1,2,4,5-tetrazin-3-yl)pyridin-3-ylamino)pentanoic acid (**9**) and (4-(6-methyl-1,2,4,5-tetrazin-3-yl)phenyl)methanol (**6**) were synthesised according to previously published procedures.[2,3]

Silica gel was treated with $EtSiCl_3$ according to a published procedure.[4] The DNA strands **s0–NH$_2$** and **s0–MAAm** were purchased from Integrated DNA Technologies Ltd. and resuspended in 18 MΩ water to a concentration of 200 μM prior to use. NAP-5 sephadex purification columns were purchased from GE Healthcare. Poly(NIPAM) containing a terminal norbornene group (**P7**) was synthesised according to a previously published procedure.[2] ZipTip® pipette tips were purchased from Merck Millipore. 4-Cyano-4-(dodecylthiocarbonothioylthio)pentanoic acid (**7**) was purchased from Sigma-Aldrich and used as received. Bio-Beads S-X1 for preparatory size exclusion chromatography were purchased from Bio-Rad Laboratories Inc.

1.2 Size exclusion chromatography (SEC)

DMF SEC data were obtained in HPLC grade DMF containing 1 mg mL^{-1} lithium bromide at 323 K, with a flow rate of 1.0 mL min^{-1}, on a set of two Varian PLgel 5 µm Mixed-D columns (7.5 mm diameter), with guard column. THF SEC data were obtained in HPLC grade THF containing 2 % triethylamine at 293 K, with a flow rate of 1.0 mL min^{-1}, on a set of two Varian PLgel 5 µm Mixed-D columns (7.5 mm diameter), with guard column. $CHCl_3$ SEC data were obtained in HPLC grade $CHCl_3$ at 293 K, with a flow rate of 1.0 mL min^{-1}, on a set of two Varian PLgel 5 µm Mixed-D columns (7.5 mm diameter), with guard column. SEC data were analysed using Cirrus SEC software calibrated using poly(methyl methacrylate) standards (690-271 400 Da) or poly(styrene) standards (162-371 100 Da).

1.3 NMR spectroscopy

^{1}H, ^{19}F and ^{13}C NMR spectra were recorded on Bruker DPX-300 or -400 spectrometers at 293 K. Chemical shifts are reported as δ in parts per million (ppm) and referenced to the residual solvent resonances ($CDCl_3$ ^{1}H: δ = 7.26 ppm; ^{13}C δ = 77.16 ppm. *d$_6$*-DMSO ^{1}H: δ = 2.50 ppm; ^{13}C: δ = 39.52 ppm).

1.4 IR spectroscopy

IR measurements were collected on a PerkinElmer Spectrum 100 FT-IR spectrometer. Solid samples were crushed and then applied to the FTIR sensor; liquid samples were applied as a small droplet.

1.5 UV-vis spectroscopy

UV-vis measurements were collected on a PerkinElmer Lambda 35 spectrometer using a Hellma TrayCell with a 1 mm path length adapter or, for cloud point measurements, with a quartz cell with a 1 cm path length. DNA solution concentrations were determined using UV-vis absorption measurements at 260 nm and the known extinction coefficient supplied by the manufacturer.

1.6 Mass spectrometry

ESI mass spectra were collected on a Bruker Esquire2000 ESI-MS machine using either methanol (for small molecules) or a 1:1 mixture of 2-propanol and 50 mM ammonium acetate (for DNA samples) as solvent. MALDI-ToF mass spectra were collected on a Bruker Ultraflex II MALDI-ToF machine using 3-hydroxypicolinic acid as the matrix.

1.7 Electrophoresis

Native polyacrylamide gel electrophoresis (PAGE) was carried out with 1 × Tris-Acetate EDTA (TAE) as running buffer at 4°C and constant voltage of 200 V, loading with glycerol/bromophenol blue loading buffer. All gels were run using a Bio-Rad Mini-Protean Tetra System apparatus, and visualised using SYBR Gold nucleic acid stain, purchased

from Invitrogen, under UV transillumination with a UVITEC UVIdoc HD2 gel documentation system. Samples were diluted so that approximately 1 pmol of DNA was added to each lane of the gel (typically 10 µL of a 100 nM solution). Yields were estimated by densitometry using the Image-J image analysis package by taking the area under the peak of interest and dividing it by the area under all DNA-containing peaks.

1 × TAE buffer consisted of 40 mM Tris-acetate and 1 mM EDTA. 1 × TE buffer consisted of 10 mM Tris-HCl and 1 mM EDTA. The native loading buffer consisted of 25 % glycerol and 0.05 % bromophenol blue in 1 × TE buffer, and was diluted five-fold before use.

1.8 HPLC

HPLC analyses were performed on a Varian 920-LC™ integrated liquid chromatography system. Chromatography was performed on a Waters XBridge™OST C18 2.5 µm 4.6 × 50 mm column heated to 40°C (for DNA strands) or 24°C (for DNA–polymer conjugate). Flow rate was set at 1 mL min^{-1} with a linear gradient of the following buffers: Buffer A, 0.1 M triethylammonium acetate, 5 % acetonitrile, pH 7.0; buffer B, 0.1 M triethylammonium acetate, 70 % acetonitrile, pH 7.0. Fractions collected were combined and concentrated using an Eppendorf concentrator plus.

2 Experimental

2.1 Synthesis of poly(NIPAM) using DDMAT

Polymers containing a carboxylic acid end-group were synthesised using the CTA DDMAT, **1**. A typical procedure was as follows. **1**(0.032 g, 0.09 mmol), NIPAM (1.000 g, 8.84 mmol) and AIBN (0.003 g, 0.02 mmol) were dissolved in 1,4-dioxane (3 mL). The solution was rigorously de-gassed by four successive freeze-pump-thaw cycles, sealed under nitrogen and heated at 65 °C for 32 hours. The reaction mixture was allowed to cool and the solvent then removed *in vacuo*. The solid was taken up in the minimum volume of THF possible and the polymer product precipitated into diethyl ether (200 mL) at room temperature. The product was isolated by filtration as a light yellow solid (0.708 g, 71 %)[1] and analysed by DMF SEC using PMMA calibration standards (M_n 10 400 Da, Đ 1.14). ^1H NMR (300 MHz, $CDCl_3$) δ 7.00-5.50 (br s, PNIPAM N*H*), 4.10-3.80 (br s, PNIPAM $NCH(CH_3)_2$), 2.40-0.73 (br m, polymer backbone *H*) ppm.

2.2 Removal of the RAFT end group using EPHP

The trithiocarbonate end group was removed from poly(NIPAM) as follows.[40] **P1a** (0.200 g, 0.01 mmol), 1-ethylpiperidine hypophosphite (EPHP) (0.060 g, 0.33 mmol) and AIBN (2 mg, 10 µmol) were dissolved in anhydrous DMF (5 mL) and transferred to an oven-dried ampoule. The solution was rigorously degassed by three successive freeze-pump-thaw cycles and then stirred under nitrogen at 100 °C for 2 hours. Water (20 mL) was added and the solution dialysed (MWCO 1 kDa) against 18 MΩ water, with five water changes. The solution was freeze-dried to yield a white powder (0.150 g, 75 %), which was analysed by SEC using DMF as the eluent and PMMA calibration standards (M_n 6 800 Da, Đ 1.06). ^1H NMR (400 MHz, d_6-DMSO) δ 11.93 (br s, end group CO_2H), 8.00-6.40 (br m, PNIPAM N*H*), 3.84 (br s, PNIPAM $NHCH(CH_3)_2$), 2.40-0.50 (br m, polymer backbone *H*) ppm.

---

[1] Percentage yields were calculated from the final monomer conversion, which was assessed by ^1H NMR spectroscopy at the end of the polymerisation.

End group removal from poly(NIPAM) samples of various molecular weights was achieved using the same method, keeping the concentration and number of equivalents of EPHP and AIBN constant.

2.3 DNA–polymer conjugation using amide coupling reagents

For a full list of the coupling agents and solvents tested, see Table S1, Table S2 and Table S3. A general procedure follows; unless otherwise stated, stock solutions were made up in the appropriate reaction solvent. The acid-functionalised polymer (1 µL, 10 mM in DMF), coupling agents (1 µL, 10 mM) and **s0–NH$_2$** (0.5 µL, 200 µM in water) were mixed and the solution topped up to 9.5 µL with the appropriate reaction solvent. DIPEA (0.5 µL, 20 mM) was added, and the mixture vortexed briefly then left overnight at room temperature. Water (70 µL) and 5 × glycerol loading buffer (20 µL) were added and the mixture analysed by 15 % native PAGE.

2.4 Synthesis of PFP–DDMAT, 2

PFP–DDMAT, **2**, was synthesised as follows. **1** (0.500 g, 1.37 mmol) was added to an oven-dried schlenk flask, which was then evacuated and refilled with nitrogen three times. Anhydrous DMF (7.5 mL) was added *via* syringe and the flask cooled to 0°C with an ice bath. DIPEA (354 µL, 2.74 mmol) was then added *via* syringe, followed by dropwise addition of pentafluorophenyl trifluoroacetate (283 µL, 1.65 mmol). After one hour stirring at 0 °C, the flask was opened to the air and diethyl ether (30 mL) was added, followed by a 1 M solution of HCl (30 mL). The organic layer was collected and washed with water (2 × 30 mL) and brine (30 mL). The solvent was removed *in vacuo* to give a yellow oily residue, which was then purified by silica gel column chromatography, eluting with a mixture of ethyl acetate and pet. ether 40-60 (gradient from 5-10 % ethyl acetate). The fractions containing the product (R_f = 0.81) were combined and the solvent removed *in vacuo* to yield CTA **2** as a yellow oil (0.686 g, 94 %). ^{1}H NMR (400 MHz, $CDCl_3$) δ 3.31 (t, *J* = 7 Hz, 2H, $SC\mathit{H}_2$), 1.86 (s, 6H, $C(C\mathit{H}_3)_2$), 1.69 (quint, *J* = 7 Hz, 2H, $SCH_2C\mathit{H}_2$), 1.40 (m, 2H, $C\mathit{H}_2CH_3$), 1.26 (br s, 16H, $SCH_2CH_2(C\mathit{H}_2)_8$), 0.88 (t, *J* = 7 Hz, 3H, $CH_2C\mathit{H}_3$) ppm. ^{13}C NMR (150 MHz, $CDCl_3$) δ 219.9 (*C*=S), 169.6 (*C*=O), 142.1 (t), 140.4 (t), 138.7 (t), 137.0 (t) (PFP *C*s), 55.4 ($\mathit{C}(CH_3)_2$), 37.2 ($S\mathit{C}H_2$), 31.9, 29.6, 29.5, 29.4, 29.3, 29.1, 29.0, 28.9, 27.8, 25.4 ($C(\mathit{C}H_3)_2$), 22.7 ($\mathit{C}H_2CH_3$), 14.1 ($S(CH_2)_{11}\mathit{C}H_3$) ppm. ^{19}F NMR (375 MHz, $CDCl_3$) −151.5 (d, 2F, ortho *F*), −157.7 (t, 2F, para *F*), −162.3 (t, 2F, meta *F*) ppm. IR (v_{max} / cm^{-1}): 2925, 2854, 1779, 1517, 1079, 992, 815. ESI HR MS calcd. for $C_{23}H_{31}F_5O_2S_3$ $[M+H]^+$ 531.1486 Da; observed 531.1480 Da.

2.5 Synthesis of NHS–DDMAT, 3

NHS–DDMAT, **3**, was synthesised as follows.[25] **1** (0.500 g, 1.37 mmol), NHS (0.158 g, 1.37 mmol) and DCC (0.283 g, 1.37 mmol) were dissolved in dichloromethane and the mixture stirred for 48 hours. The cloudy mixture was filtered through a 0.45 µm PTFE syringe filter and the retentate washed with CH_2Cl_2 (5 mL). This process was then repeated. The solvent was removed *in vacuo* and the residue dissolved in a small amount of ethyl acetate. The solution was purified by silica gel column chromatography, eluting with a mixture of ethyl acetate and pet. ether 40-60 (1:1). The product fractions (R_f = 0.53) were collected and the solvent removed *in vacuo* to afford **3** as a yellow solid (0.502 g, 79 %). ^{1}H NMR (400 MHz, $CDCl_3$) δ 3.30 (t, *J* = 7 Hz, 2H, $SC\mathit{H}_2$), 2.81 (br s, 4H, $C\mathit{H}_2(C{=}O)N$), 1.87 (s, 6H, $C(C\mathit{H}_3)_2$), 1.68 (quint, *J* = 7 Hz, 2H, $SCH_2C\mathit{H}_2$), 1.38 (m, 2H, $C\mathit{H}_2CH_3$), 1.25 (br s, 16H, $SCH_2CH_2(C\mathit{H}_2)_8$), 0.88 (t, *J* = 7 Hz, 3H, $CH_2C\mathit{H}_3$) ppm. ^{13}C NMR (150 MHz, $CDCl_3$) δ 218.8 (*C*=S), 169.1 (*C*=OO), 168.7 (NHS *C*=O), 54.3 ($\mathit{C}(CH_3)_2$), 37.2 ($S\mathit{C}H_2$), 31.9 ($SCH_2\mathit{C}H_2$), 29.6, 29.5, 29.4, 29.3, 29.1, 29.0, 27.8, 25.4 (NHS $\mathit{C}H_2$), 25.3

($C(CH_3)_2$), 22.7 (CH_2CH_3), 14.1 ($S(CH_2)_{11}CH_3$) ppm. IR (ν_{max} / cm^{-1}): 2917, 2848, 1777, 1736, 1203, 1074, 811. ESI HR MS calcd. for $C_{21}H_{35}NO_4S_3$ $[M+Na]^+$ 484.1626 Da; observed 484.1619 Da.

2.6 Synthesis of poly(NIPAM) using NHS– and PFP–DDMAT

Polymerisation of NIPAM with **3** was conducted as follows. NHS–DDMAT, **3**, (0.041 g, 0.09 mmol), NIPAM (1.000 g, 8.84 mmol) and AIBN (0.002 g, 0.01 mmol) were dissolved in 1,4-dioxane (1.5 mL) and transferred to an oven-dried ampoule. The mixture was subjected to three freeze-pump-thaw cycles and sealed under an atmosphere of nitrogen. It was then placed in an oil bath preheated to 65 °C. After 2 hours the ampoule was removed and the reaction quenched by opening it to the air and cooling with liquid nitrogen. The solution was poured into pet. ether 40-60 (80 mL) cooled in an ice bath and the precipitant collected by filtration. The product was then dissolved in THF (1 mL) and the process repeated 5 more times. Finally, the isolated solid was dissolved in THF (1 mL) and precipitated into diethyl ether (80 mL) cooled in an ice bath. The product was isolated by filtration, dried *in vacuo* and isolated as a yellow powder (0.335 g, 36 %) and analysed by DMF SEC using PMMA calibration standards (M_n 9 610 Da, Đ 1.10). ^1H NMR (400 MHz, $CDCl_3$) δ 7.36-5.55 (br m, PNIPAM N*H*), 4.00 (br s, PNIPAM C*H*(CH_3)$_2$), 3.33 (br m, 2H, SC*H*$_2$), 2.86 (br s, 4H, C*H*$_2$(C=O)N), 2.64-0.80 (br m, PNIPAM backbone *H*), 0.88 (t, *J* = 7 Hz, 3H, $S(CH_2)_{11}CH_3$) ppm.

PFP–DDMAT, **2**, was also used to polymerise NIPAM using an identical procedure. The product was collected as a yellow solid (0.266 g, 32 %) and analysed by DMF SEC using PMMA calibration standards (M_n 7 760 Da, Đ 1.10). ^1H NMR (400 MHz, $CDCl_3$) δ 7.40-5.70 (br m, PNIPAM N*H*), 4.00 (br s, PNIPAM C*H*(CH_3)$_2$), 3.35 (br m, 2H, SC*H*$_2$), 2.65-0.75 (br m, PNIPAM backbone *H*), 0.88 (t, *J* = 7 Hz, 3H, $S(CH_2)_{11}CH_3$) ppm. ^{19}F NMR (375 MHz, $CDCl_3$) δ −153.0 (br m, 2F, PFP end group F_{ortho}), −158.0 (br m, 1F, PFP end group F_{para}), −162.3 (br m, 2F, PFP end group F_{meta}) ppm.

2.7 Removal of the trithiocarbonate group using AIBN and LPO

P2 (100 mg, 0.01 mmol), AIBN (187 mg, 1.14 mmol) and LPO (18 mg, 0.05 mmol) were dissolved in dry toluene (28 mL) and the solution degassed by three freeze-pump-thaw cycles and then sealed under nitrogen. The mixture was heated to 80 °C for five hours, then allowed to cool to room temperature. The solvent was removed *in vacuo* and the residue resuspended in THF (1 mL), which was then poured into pet. ether 40-60 (15 mL) cooled with an ice bath. The precipitated product was collected by filtration and dried under vacuum to give a white powder (71 mg, 71 %), which was analysed by DMF SEC using PMMA calibration standards (M_n 8 170 Da, Đ 1.11). ^1H NMR (400 MHz, $CDCl_3$) δ 8.00-5.50 (br m, PNIPAM N*H*), 4.00 (br s, PNIPAM C*H*(CH_3)$_2$), 3.00-0.50 (br m, PNIPAM backbone *H*) ppm. ^{19}F NMR (375 MHz, $CDCl_3$) δ −153.0 (br m, 2F, PFP end group F_{ortho}), −158.0 (br m, 1F, PFP end group F_{para}), −162.3 (br m, 2F, PFP end group F_{meta}) ppm.

The trithiocarbonate group was removed from **P3** using the same method to yield a white powder (61 mg, 61 %), which was analysed by DMF SEC using PMMA calibration standards (M_n 9 780 Da, Đ 1.11). ^1H NMR (400 MHz, $CDCl_3$) δ 7.40-5.40 (br m, PNIPAM N*H*), 4.00 (br s, PNIPAM C*H*(CH_3)$_2$), 2.86 (br s, 4H, C*H*$_2$(C=O)N), 2.60-0.80 (br m, PNIPAM backbone *H*) ppm.

2.8 Attempted conjugation of P2 and P3 to s0–NH$_2$

The reaction solvents tested were: DMF, DMAc, NMP and MeCN. The polymer (9.5 μL, 10, 100 or 1000 μM in the reaction solvent) was mixed with **s0–NH$_2$** (0.5 μL, 200 μM in

water) and left overnight at room temperature. 5 × glycerol loading buffer (20 μL) and water (70 μL) were added and the reaction mixture analysed by 15 % native PAGE.

2.9 Polymer end group removal using $NaBH_4$

The trithiocarbonate group present in **P1a-c** was reduced using a previously reported procedure.[41] An example procedure follows. **P1a** (0.200 g, 0.03 mmol) was dissolved in water (20 mL) and sodium borohydride (0.378 g, 10.00 mmol) was added. The mixture was stirred at room temperature for 2.5 hours with vigorous stirring, then dialysed against 18 MΩ water for 4 days incorporating 5 water changes. The solution was then freeze-dried to obtain the thiol-terminated product (**P4a**) as a white solid (0.131 g, 66 %) and analysed by DMF SEC using PMMA calibration standards (M_n 5 740 Da, Đ 1.17). ^{1}H NMR (400 MHz, $CDCl_3$) δ 7.20-5.50 (br s, PNIPAM N*H*), 3.99 ppm (br s, PNIPAM NC*H*(CH_3)$_2$), 3.20-0.50 (br m, polymer backbone *H*) ppm. **P4b** and **P4c** were synthesised using an identical procedure.

2.10 Ellman's assay

Ellman's assay was carried out as follows. Ellman's reagent (0.4 mg mL^{-1}) was dissolved in potassium phosphate buffer solution (100 mM, pH 8.0) to give Ellman's solution. The polymer under investigation (1 mg) was dissolved in water (600 μL) which had been purged with nitrogen for 30 minutes. This solution was then diluted with 1.2 mL of potassium phosphate buffer solution (100 mM, pH 8.0). Finally, 200 μL of Ellman's solution was added and the solution mixed. The absorbance at 412 nm was recorded and the concentration of thiol determined by using the extinction coefficient of Ellman's solution (ε_{412} = 14 150 M^{-1} cm^{-1}). Finally, the percentage incorporation of thiol was calculated by comparing the calculated concentration to the theoretical concentration (which assumes one thiol group per polymer chain).

2.11 Conjugation of P4a-c to s0–MAAm without catalyst

s0–MAAm (0.5 μL, 200 μM in water, 0.1 nmol) was added to thiol-terminated poly(NIPAM) (**P4a-c**) (10 μL, 1 mM in the reaction solvent) and the mixture shaken for 24 hours at 40 °C. The reaction mixture was then analysed by 15 % native PAGE. The reaction was also attempted with fewer equivalents of polymer and at room temperature.

2.12 Conjugation of poly(NIPAM)–SH to s0–MAAm with TCEP catalyst

s0–MAAm (0.5 μL, 200 μM in water, 0.1 nmol) was added to a 10 μL solution of TCEP (10 mM) and thiol-terminated poly(NIPAM) (**P4a-c**) (1 mM) and the solution shaken at 40 °C for 24 hours. The reaction mixture was then analysed by 15 % native PAGE.

2.13 *In situ* aminolysis of P5 in the presence of s0–MAAm

s0–MAAm (0.57 μL, 173 μM in water, 0.1 nmol) was mixed with hexylamine (4 μL, 100 mM in the reaction solvent), TEA (4 μL, 100 mM in the reaction solvent),**P5** (1 μL, 10 mM in the reaction solvent, 10 nmol) and the reaction solvent (0.43 μL, MOPS pH 8.0, DMF or DMSO). After being left at room temperature overnight under an atmosphere of nitrogen, the reaction mixture was analysed by 15 % native PAGE.

2.14 Conjugation of P4a-c to s0–MAAm with DMPP catalyst

s0–MAAm (0.58 μL, 173 μM in water, 0.1 nmol) was mixed with DMPP (5 μL, various concentrations in the reaction solvent) and thiol-terminated poly(NIPAM) (**P4a-c**) (5 μL, various concentrations in the reaction solvent). The solution was left for 24 hours at room temperature under an atmosphere of nitrogen and then analysed by 15 % native PAGE,

which revealed the formation of the DNA–polymer conjugate in approximately 5 % yield (as assessed by densitometry).

2.15 Synthesis of s0–AAm

Acrylamide-functionalised DNA (**s0–AAm**) was synthesised from amine-functionalised DNA (**s0–NH$_2$**) as follows. Acrylic acid (16.7 µL, 600 mM in DMF, 10 µmol), EDCI (16.7 µL, 600 mM in DMF, 10 µmol), HOBt (16.7 µL, 600 mM in DMF, 10 µmol) and DIPEA (1.7 µL, 10 µmol) were mixed and incubated at room temperature for thirty minutes. Phosphate buffer pH 8.0 (47.0 µL) and **s0–NH$_2$** (3.2 µL, 3.17 mM in water, 10 nmol) were added and the mixture left at room temperature for 24 hours. The excess small molecules and DMF were removed by extraction with dichloromethane (3 × 200 µL). The aqueous layer was isolated and topped up to a final volume of 100 µL with water. The product was isolated by HPLC, with a yield of 20 % as quantified by UV-vis spectroscopy using the known extinction coefficient of the starting material DNA at 260 nm.

2.16 Conjugation of P4a-c to s0–AAm

Conjugation of thiol-terminated poly(NIPAM) (**P4a-c**) to **s0–AAm** was attempted using identical conditions to those employed above for **s0–MAAm**. No product was observed under any of the conditions used.

2.17 Synthesis of s0–Mal using the bifunctional adapter, 5

s0–NH$_2$ (1000 µL, 200 µM in water, 200 nmol), **5** (53.6 mg, 200 mmol) and DIPEA (35 µL, 200 mmol) were mixed in DMF (1000 µL) and the reaction shaken overnight at 40 °C. The excess small molecules were then removed using a NAP-10 Sephadex column and the collected solution concentrated *in vacuo* and purified by HPLC. The product was isolated as a single fraction, with an isolated yield of 63 % as quantified by UV-vis spectroscopy using the known extinction coefficient of the starting material DNA at 260 nm.

2.18 Conjugation of P4a-c to s0–Mal

s0–Mal (25 µL, 20 µM in MOPS buffer pH 8.0, 0.5 nmol) was mixed with thiol-terminated poly(NIPAM) (**P4a-c**) (25 µL, various concentrations in MOPS buffer pH 8.0) and the solution incubated at 40 °C for 24 hours. Analysis of the reaction mixtures by 15 % native PAGE revealed that the DNA–polymer conjugate (visible as a broad, low-mobility band) had been formed in up to 58 % yield.

2.19 Conjugation of P4a-c to s0–Mal in organic solvents

s0–Mal (2 µL, 50 µM in water, 0.1 nmol) was mixed with **P4a-c** (1 µL, 10 mM in the reaction solvent) and DIPEA (0.5 µL, 20 mM in the reaction solvent) and the reaction solvent (DMF, THF, NMP, DMSO or MeCN – 6.5 µL). The solution was left for 24 hours at room temperature and then analysed by 15 % native PAGE. The reaction mixture was also analysed by HPLC to assess the degree of degradation of the maleimide group.

2.20 RAFT polymerisation using CTA 4

NIPAM was polymerised using the alkyne-functionalised CTA, **4**, as follows. NIPAM (1 g, 8.84 mmol), **4** (29 mg, 0.09 mmol) and AIBN (3 mg, 0.02 mmol) were dissolved in DMF (2 mL) and the mixture transferred to an oven-dried ampoule under nitrogen. The solution was degassed by four successive freeze-pump-thaw cycles, then sealed under nitrogen and heated to 65 °C for 23 hours. The solution was concentrated *in vacuo* and then poured into diethyl ether (300 mL). The precipitated product was isolated by filtration as a light pink solid (0.32 g, 51 %), and analysed by DMF SEC using PMMA calibration standards (M_n 6.9 kDa, Đ 1.16). ^{1}H NMR (400 MHz, $CDCl_3$) δ 7.94 (t, 2H,

polymer end group Ar*H*), 7.54 (t, 1H, polymer end group Ar*H*), 7.37 (t, 2H, polymer end group Ar*H*), 7.32-7.14 (br m, polymer end group Ar*H*), 7.10-5.40 (br s, PNIPAM N*H*), 4.82-4.44 (br m, 2H, polymer end group CH_2C≡CH), 4.25-3.70 (br s, PNIPAM C*H*(CH_3)$_2$), 3.59 (br s, 1H, polymer end group C≡C*H*), 2.60-0.80 (br m, PNIPAM backbone *H*) ppm.

2.21 Synthesis of s0–Nb

EDCI (100 µL, 300 mM in DMF) was mixed with HOBt (100 µL, 300 mM in DMF), 5-norbornene-2-carboxylic acid (100 µL, 300 mM in DMF) and PBS (150 µL) and thoroughly mixed. 75 µL of this solution was mixed with **s0–NH_2** (25 µL, 200 µM in water) and DIPEA (0.87 µL). After one hour shaking the flask at room temperature the mixture was purified by HPLC and the product isolated as two separate peaks. Both were analysed by MALDI-ToF MS. Expected mass 6 996.3 Da; observed 6 995.8.

2.22 Reaction of s0–Nb with 6

The DNA strand **s0–Nb** (14.3 µL, 7 µM in water) was added to a centrifuge tube and the solvent removed *in vacuo*. HPLC buffer (9 µl, 100 mM TEAA, 70 % MeCN) was added and then **6** (1 µL, 10 mM in DMSO). After twenty-four hours the mixture was purified by ZipTip to remove excess small molecules, and analysed by HPLC, which revealed a significant peak shift from the starting material.

2.23 Attempted modification of PFP-containing P2 with 6

Post-polymerisation modification of a polymer synthesised using CTA **2** was attempted as follows. Poly(NIPAM) (20 mg, 1 µmol), **6** (2 mg, 10 µmol) and TEA (0.5 mg, 5 µmol) were dissolved in anhydrous THF (0.25 mL) and stirred under nitrogen at 35 °C for two hours, then for a further fifteen hours at room temperature. The solution was purified by preparatory SEC (Bio-Beads S-X1) and the polymer isolated, dried and analysed by ^{1}H NMR spectroscopy and DMF SEC. Both indicated that no reaction had taken place.

2.24 Perfluorophenyl 4-cyano-4-(dodecylthiocarbonothioylthio)-pentanoate, 8

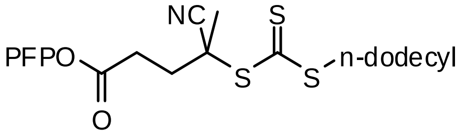

Perfluorophenyl 4-cyano-4-(dodecylthiocarbonothioylthio)pentanoate (**8**) was synthesised as follows.[38] **7** (0.5 g, 1.24 mmol) was added to an oven-dried flask under nitrogen. Anhydrous DMF (9 mL) was added followed by DIPEA (431 µL, 2.48 mmol). The mixture was cooled using an ice bath and pentafluorophenyl trifluoroacetate (255 µL, 1.49 mmol) was added dropwise with vigorous stirring. The reaction was allowed to proceed for one hour, then diethyl ether (40 mL) was added followed by 1 M HCl (40 mL). The organic layer was separated and washed with water (2 × 40 mL) and brine (40 mL). The solvent was then removed *in vacuo* and the residue purified by silica gel column chromatography, eluting with a mixture of hexane and diethyl ether (10:1). The product (R_f 0.21) was isolated as an orange viscous liquid (0.563 g, 80 %). ^{1}H NMR (300 MHz, $CDCl_3$) δ 3.34 (t, J = 7 Hz, 2H, SCH_2), 3.01 (m, 2H, CH_2CO_2PFP), 2.60 (m, 2H, C$H_2CH_2CO_2$PFP), 1.93 (s, 3H, SCCH_3), 1.70 (quint, J = 7 Hz, 2H, SCH_2CH_2), 1.40 (m, 2H, SCH_2CH_2CH_2), 1.26 (br s, 16H, S$(CH_2)_3$(CH_2)$_8CH_3$), 0.88 (t, J = 7 Hz, 3H, S$(CH_2)_{11}$CH_3) ppm. IR (ν_{max} / cm^{-1}) 2918, 2850, 1797, 1517, 1094, 990, 803. (These values compare well with the literature values above, but are given because a significantly different method was used for the synthesis.)

2.25 RAFT polymerisation of NIPAM using CTA 8

NIPAM (1 g, 8.84 mmol), **8** (50.3 mg, 0.09 mmol) and AIBN (1.5 mg, 0.01 mmol) were dissolved in 1,4-dioxane (2 mL) and the mixture degassed by three successive freeze-pump-thaw cycles. After warming to room temperature, the reaction vessel was sealed under nitrogen and placed in an oil bath heated to 65 °C for four hours. The solution was diluted with THF (1 mL) and the polymer precipitated into diethyl ether (300 mL) cooled with an external dry ice bath. The solvent was decanted and the solid re-dissolved in THF (2 mL) and precipitated into cold diethyl ether once more. The product was isolated by filtration and drying as a pale yellow solid (841 mg, 84 %[2]), which was analysed by DMF SEC using PMMA calibration standards (M_n 13.0 kDa, Đ 1.05). ^{1}H NMR ($CDCl_3$, 400 MHz) δ 7.40-5.50 (br m, PNIPAM N*H*), 3.93 (br s, PNIPAM NHC*H*), 3.26 (br m, 2H, SC*H*$_2$), 2.80 (br m, 2H, C*H*$_2$CO$_2$PFP), 2.50-0.50 (br m, PNIPAM backbone *H* and NCH(C*H*$_3$)$_2$), 0.81 (t, *J* = 6 Hz, 3H, S(CH$_2$)$_{11}$CH$_3$) ppm. ^{19}F NMR ($CDCl_3$, 375 MHz) δ −152.6 (br s, 2F, ortho *F*), −157.6 (br s, 1F, para *F*), −162.1 (br s, 2F, meta *F*) ppm.

2.26 Modification of P6 with 6

P6 (50.0 mg, 4 μmol), **6** (8.1 mg, 40 μmol) and DMAP (0.2 mg, 2 μmol) were dissolved in THF (0.25 mL) and the solution stirred under an atmosphere of nitrogen for twenty-four hours at room temperature. The solvent was then removed by blowing with compressed air and DMF (0.5 mL) was added. The polymer was then purified from excess small molecules by preparatory SEC (Bio-Beads S-X1) using DMF as the eluent. The fastest-eluting pink band was collected and dried down to yield the product as a light pink solid (41 mg, 82 %), which was analysed by DMF SEC using PMMA calibration standards (M_n 13.1 kDa, Đ 1.04). The incorporation of the Tz group was calculated to be 53 % by comparison of the ArC*H*$_2$O peak to the polymer NHC*H* peak in the ^{1}H NMR spectrum. ^{1}H NMR ($CDCl_3$, 400 MHz) δ 8.60 (d, *J* = 8 Hz, 0.95H, Tz aromatic *H*), 7.57 (d, *J* = 7 Hz, 1.19H, Tz aromatic *H*), 7.20-5.60 (br m, PNIPAM N*H*), 5.23 (d, *J* = 6 Hz, 1.09H, CO$_2$C*H*$_2$Ar), 4.00 (br s, PNIPAM NHC*H*), 3.34 (br m, 2H, SC*H*$_2$), 3.11 (s, 1.60H, Tz-C*H*$_3$), 2.65-0.55 (br m, PNIPAM backbone *H* and NHCH(C*H*$_3$)$_2$), 0.88 (t, *J* = 7 Hz, 3H, S(CH$_2$)$_{11}$C*H*$_3$) ppm.

2.27 Conjugation of s0–Nb to P7

s0–Nb (0.5 μL, 70 μM in water) was mixed with the reaction solvent (5.0 μL) and **P7** (0.5 μL, 14 mM in DMF) and left at room temperature for forty-eight hours. The mixture was diluted with water (35 μL) and 5 × glycerol loading buffer (10 μL) and analysed by 15 % native PAGE. The yield was calculated by densitometry after staining with SYBR Gold and visualisation under UV transillumination.

[2] Based on 99 % monomer conversion as assessed by ^{1}H NMR spectroscopy at the end of the polymerisation.

2.28 2,5-dioxopyrrolidin-1-yl 5-oxo-5-(6-(6-(pyridin-2-yl)-1,2,4,5-tetrazin-3-yl)pyridin-3-ylamino)pentanoate, 10

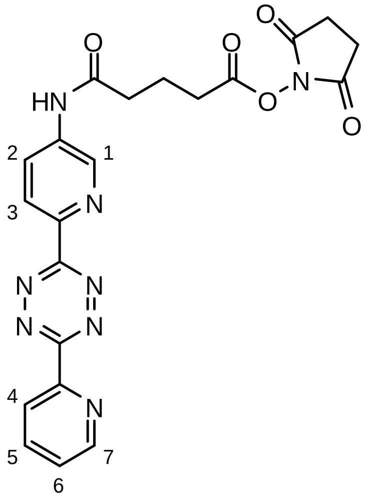

The tetrazine DNA adaptor was synthesised as follows. Tetrazine **9** (0.100 g, 0.27 mmol) and NHS (0.032 g, 0.27 mmol) were dissolved in DMF (4 mL) and the solution bubbled with nitrogen for 30 minutes. The mixture was then cooled using an ice bath and DCC (0.057 g, 0.27 mmol) dissolved in DMF (1 mL) was added via syringe. After 30 minutes stirring the ice bath was removed and the reaction stirred under nitrogen for 16 hours. The reaction mixture was adsorbed onto silica which had been previously treated with $EtSiCl_3$. $EtSiCl_3$-treated silica gel column chromatography was then performed, eluting first with DMF, then acetone. The pure fractions were collected and combined, then dried *in vacuo* to afford the product **10** as a deep red powder (0.048 g, 38 %). ^{1}H NMR (400 MHz, d_6-DMSO) δ 10.67 (br s, 1H, N*H*C=O), 9.10 (d, *J* = 2 Hz, 1H, Tz *H*1), 8.97 (d, *J* = 4 Hz, 1H, Tz *H*7), 8.66 (d, *J* = 9 Hz, 1H, Tz *H*4), 8.63 (d, *J* = 8 Hz, 1H, Tz *H*3), 8.47 (dd, *J* = 2, 9 Hz, 1H Tz *H*2), 8.19 (td, *J* = 1, 4 Hz, 1H, Tz *H*6), 7.76 (dd, *J* = 5, 7 Hz, 1H, Tz *H*5), 2.87 (m, 6H, C*H*$_2$(C=O)N and C*H*$_2$(C=O)NH), 2.63 (t, *J* = 7 Hz, 2H, C*H*$_2$(C=O)OSu), 2.03 (quint, *J* = 7 Hz, 2H, C*H*$_2$CH$_2$(C=O)NH) ppm. ^{13}C NMR (125 MHz, d_6-DMSO) δ 172.5 (*C*=ONH), 170.7 (NHS *C*=O), 169.3 (*C*=OO), 163.5 (Tz N-*C*=N), 163.2 (Tz N-*C*=N), 151.1 (Tz *C*7), 150.7 (Tz C-*C*=N), 144.3 (Tz C-*C*=N), 141.8 (Tz *C*1), 138.9 (Tz *C*-NHC=O), 138.3 (Tz *C*5), 127.0, 126.7, 125.4, 124.7 (Tz *C*2/3/4/6), 35.1 (*C*H$_2$C=ONH), 30.0 (*C*H$_2$C=OO), 25.9 (NHS *C*H$_2$), 20.2 (*C*H$_2$CH$_2$C=O) ppm. IR (ν_{max} / cm^{-1}): 2895, 1732, 1714, 1543, 1392, 1061. ESI HR MS calcd. for $C_{21}H_{18}N_8O_5$ [M+Na]$^+$ 485.1298; observed 485.1293.

2.29 Synthesis of s0–Tz

300 mM solutions of EDCI, HOBt and **10** were prepared in DMF and then mixed in equal proportions. 100 μL of **s0–NH$_2$** (200 μM in water) were added to a 1 mL centrifuge tube and the solvent removed *in vacuo*. 100 μL of the EDCI/HOBt/**10** mixture were added, followed by 100 μL of potassium phosphate buffer (100 mM, pH 8.0). The solution was vortexed to mix and then heated at 40 °C for four hours, after which time small molecules were removed by passing the solution through a NAP-5 sephadex column, eluting with water. The sample was concentrated *in vacuo* and then purified by HPLC. The product was isolated as a single peak (6 %) and analysed by LC-MS. Expected mass 7 223.9 Da; observed 7 223.2 Da.

2.30 Conjugation of s0–Tz to P8

P8 (5 µL, 20/200/2000 µM in the reaction solvent) was mixed with **s0–Tz** (3.18 µL, 31.4 µM in water) and the reaction solvent (1.82 µL). After 48 hours, the mixture was diluted with 5 × glycerol loading buffer and analysed by 15 % native PAGE. The conjugate was observed as a broad low mobility band in up to 50 % yield (calculated by densitometry).

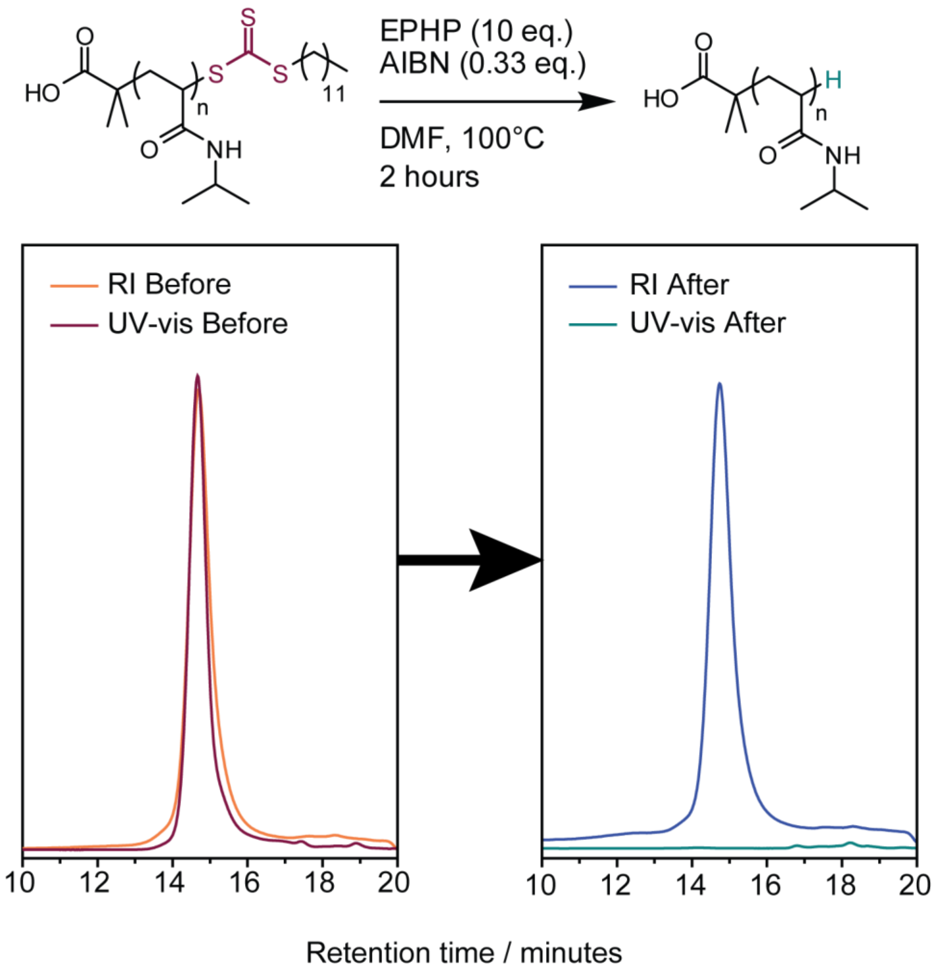

Figure S1 Removal of the trithiocarbonate group from **P1a-c** using 1-ethylpiperidine hypophosphite (EPHP) and AIBN. Left panel: UV-vis (309 nm) and RI SEC traces before end group removal. Right panel: SEC traces after end group removal.

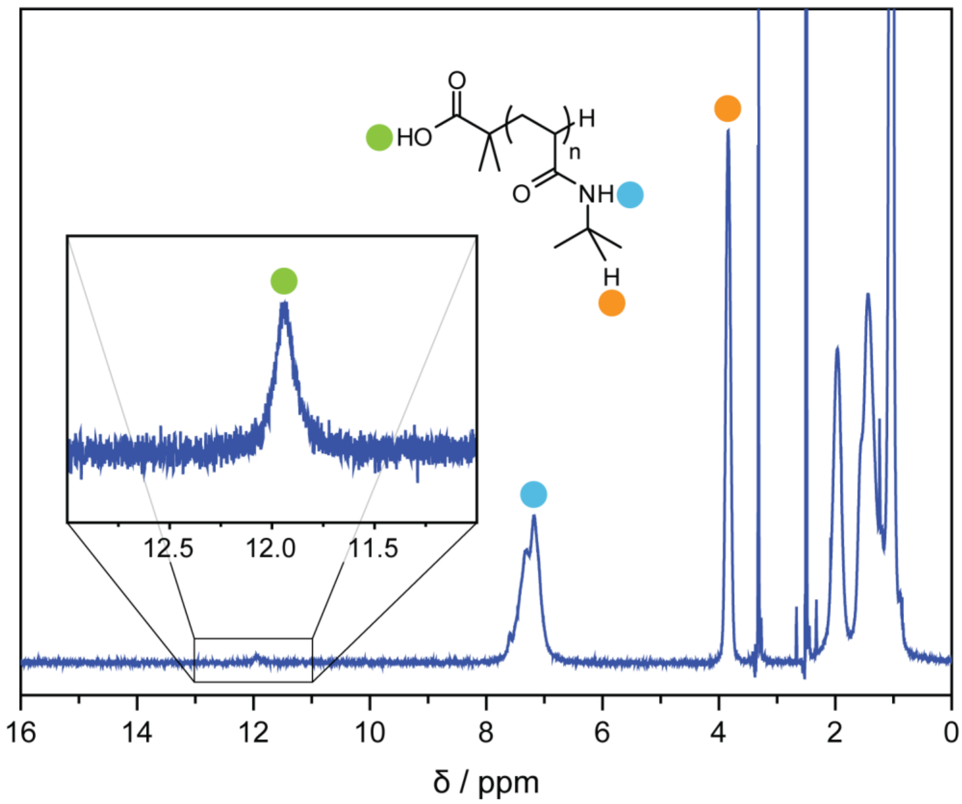

Figure S2 ¹H NMR spectrum confirming the presence of the carboxylic acid after the removal of the trithiocarbonate group from **P1a** using EPHP. Solvent: d_6-DMSO.

```
s0-NH2 =  5'- H2N-GCC CGA AAT ACC CCG TTA GAA A -3'
```

Figure S3 Base sequence of the DNA strand **s0–NH₂** used in this work.

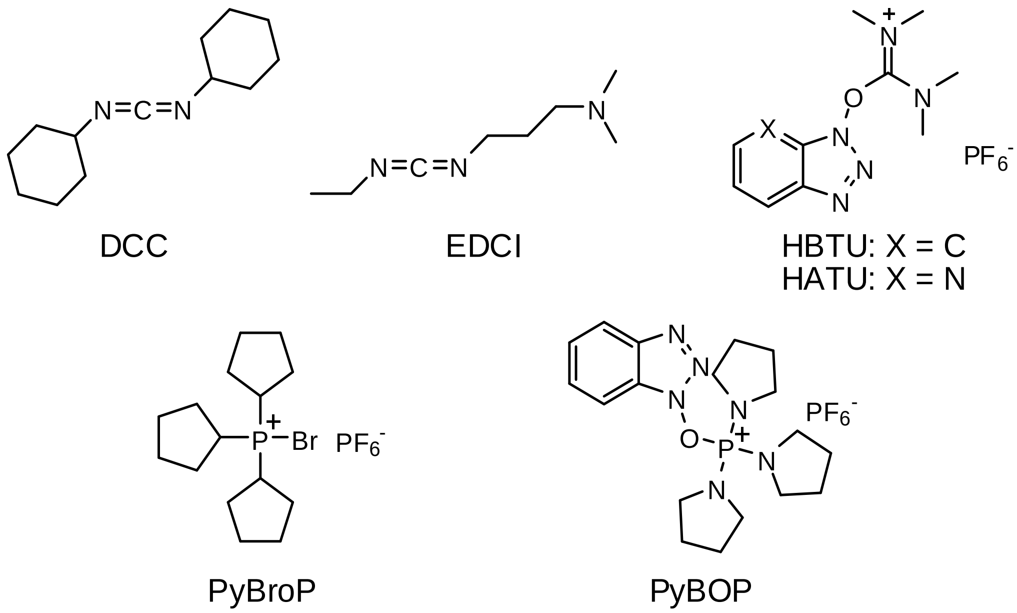

Figure S4 Structures of the coupling agents used in this study.

Table S1 Coupling agents and solvents tested for the conjugation of **P1a** to **s0–NH$_2$**. A * indicates that the coupling agent was not highly soluble in the solvent used. 100 equivalents of *N,N*-diisopropylethylamine (DIPEA) as an auxiliary base were used in all cases.

Reaction #	Coupling agent	Solvent
1a		DMF
1b*		THF
1c	EDCI/HOBt	MeCN
1d*		NMP
1e		DMSO
2a		DMF
2b		THF
2c	DCC/HOBt	MeCN
2d		NMP
2e		DMSO

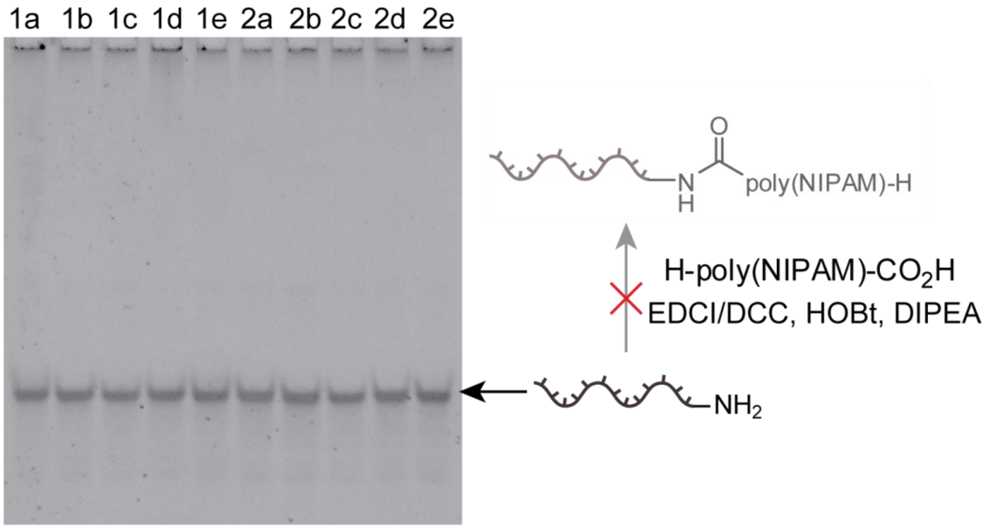

Figure S5 15 % native PAGE analysis of the DNA–polymer conjugation reactions detailed in Table S1, employing DCC and EDCI as amide coupling reagents. The only band observed was due to the starting material **s0–NH$_2$**, indicating that the expected product had failed to form.

Table S2 Further coupling agents and solvents tested for the conjugation of **P1a-c** to **s0–NH2**. A * indicates that the coupling agent was not highly soluble in the solvent used. 100 equivalents of DIPEA as auxiliary base were used in all cases.

Reaction #	Coupling agent	Solvent
3a		DMF
3b*		THF
3c	HBTU	MeCN
3d*		NMP
3e		DMSO
4a		DMF
4b*		THF
4c	HATU	MeCN
4d		NMP
4e		DMSO

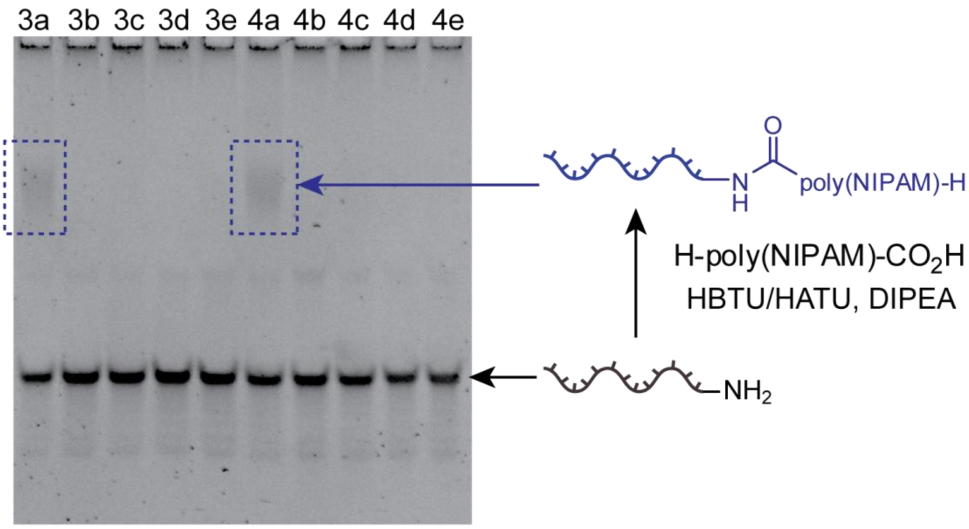

Figure S6 15 % native PAGE analysis of the DNA–polymer conjugation reactions detailed in Table S2, employing HBTU and HATU as amide coupling reagents. The band due to the starting material s0–NH2 was still the main band observed; however, in the case of reactions 3a and 4a another, slow-migrating band was also observed, which was attributed to the DNA–polymer conjugate.

Table S3 Highly active coupling agents and solvents tested for the conjugation of **P1a-c** to **s0–NH$_2$**. 100 equivalents of DIPEA as auxiliary base were used in all cases.

Reaction #	Coupling agent	Solvent
5a		DMF
5b		THF
5c	PyBOP	MeCN
5d		NMP
5e		DMSO
6a		DMF
6b		THF
6c	PyBroP	MeCN
6d		NMP
6e		DMSO

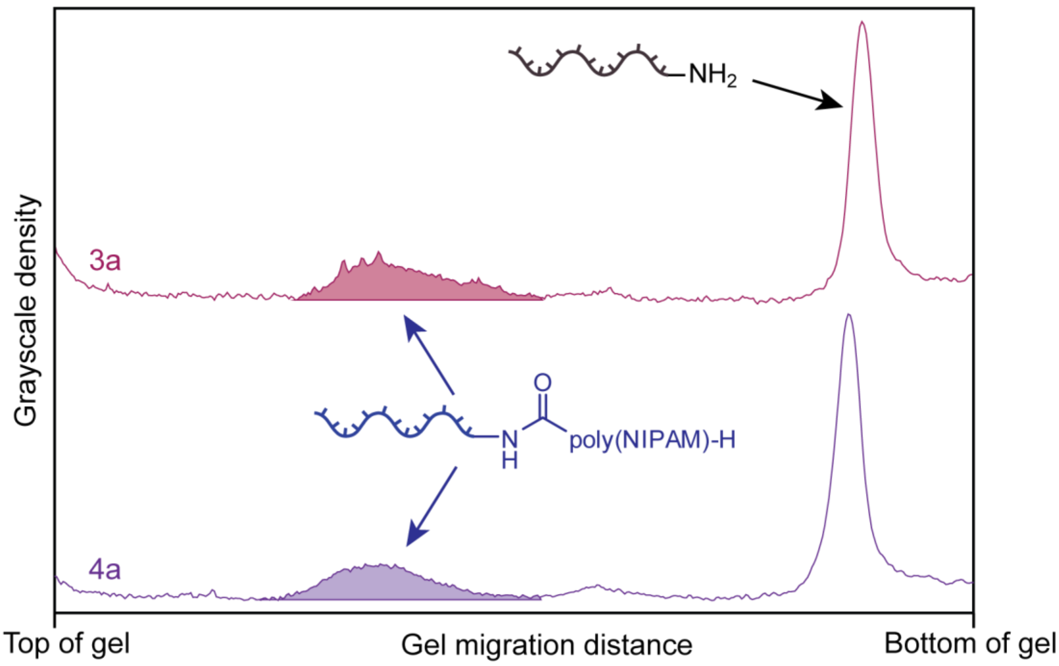

Figure S7 Densitometric analysis of lanes 3a and 4a in Figure S6. The DNA–polymer conjugate was clearly visible as a low-mobility band.

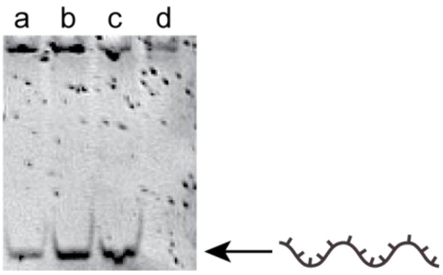

Figure S8 15 % Native PAGE analysis of control mixtures of **s0** and **s0–Mal** with **P4**. Lanes: a) **s0–Mal** exposed to reaction solvent (MOPS pH 8); b) unfunctionalised **s0**; c) unfunctionalised **s0** + **P4**; d) **P4**. No low mobility bands were observed, indicating that these are unlikely to be due to degradation products, binding of the PAGE dye by the polymer, or non-specific association of the DNA with the polymer.

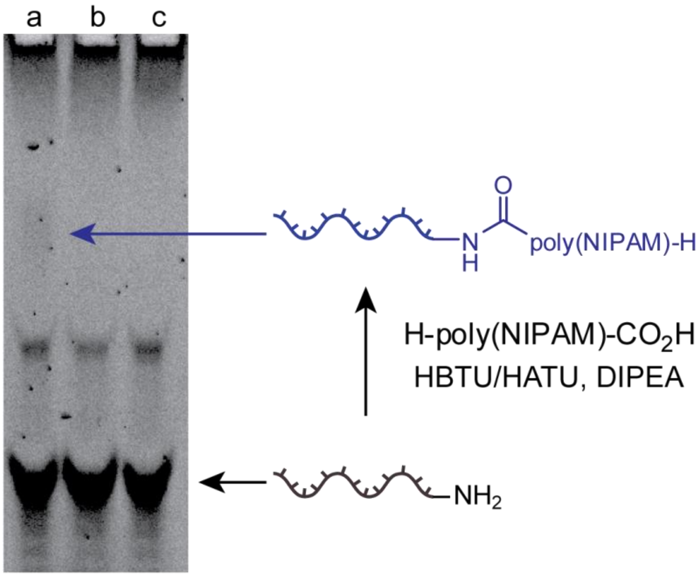

Figure S9 15 % native PAGE analysis of attempted conjugation of **P1a-c** to **s0–NH2**. Polymers used were as follows: lane a – **P1a**; lane b – **P1b**; lane c – **P1c**. A very low yield (~5 %) of the conjugate was visible as a faint band in lane a only.

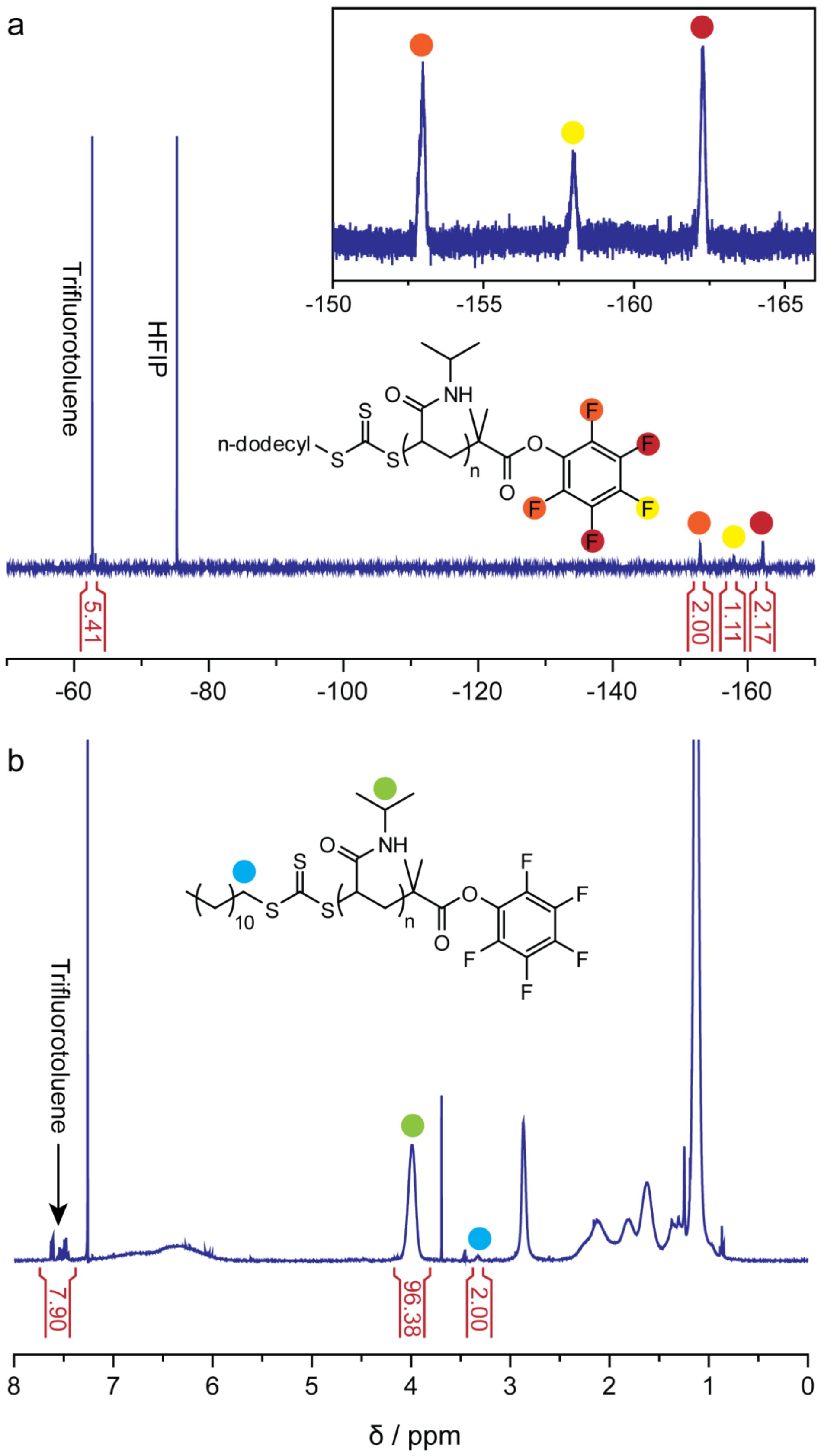

Figure S10 a) ^{19}F and b) ^{1}H NMR spectra of poly(NIPAM) containing a PFP activated ester (**P2**). Incorporation of the PFP group was quantified by comparing the integrals of, a) the fluorine signals of the PFP group, and, b) the hydrogen signals of the CH_2 group adjacent to the trithiocarbonate, with an external standard (trifluorotoluene). Solvent: $CDCl_3$.

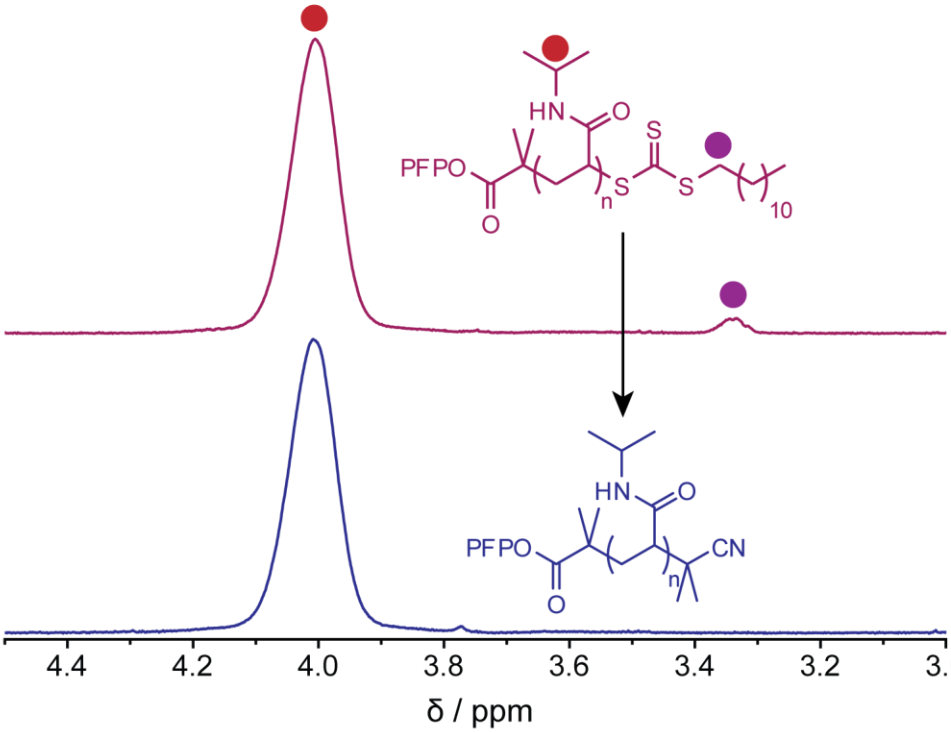

Figure S11 ^{1}H NMR spectrum showing the substitution of the trithiocarbonate group of **P2** using AIBN and LPO. Completion of the reaction is indicated by complete loss of the signal at around 3.3 ppm, which is attributed to the CH_2 group adjacent to the trithiocarbonate. Solvent: $CDCl_3$.

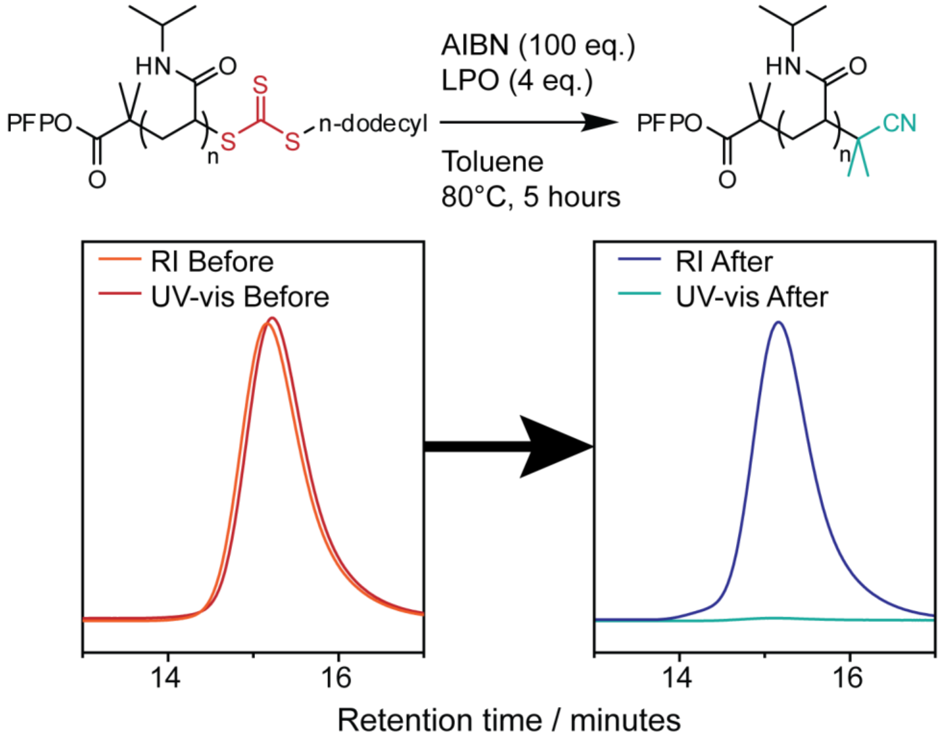

Figure S12 DMF SEC traces showing the loss of the signal at 309 nm (due to the trithiocarbonate group) upon substitution of the end group of **P2** with AIBN. In both chromatograms the UV-vis trace was scaled by the same amount relative to the RI.

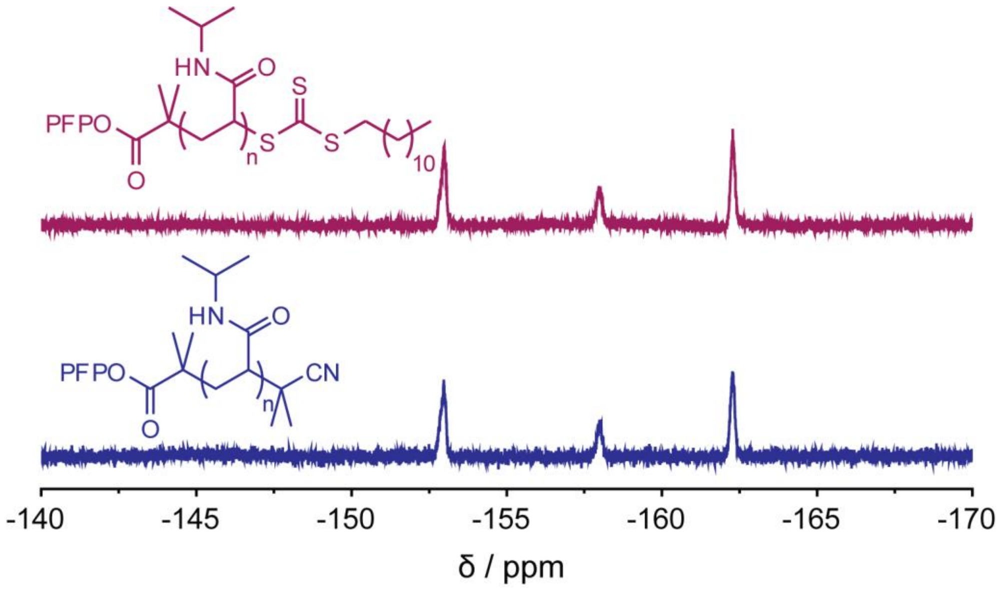

Figure S13 ^{19}F NMR spectrum showing the retention of the PFP activated ester group upon removal of the trithiocarbonate from **P2** using AIBN and LPO. Solvent: $CDCl_3$.

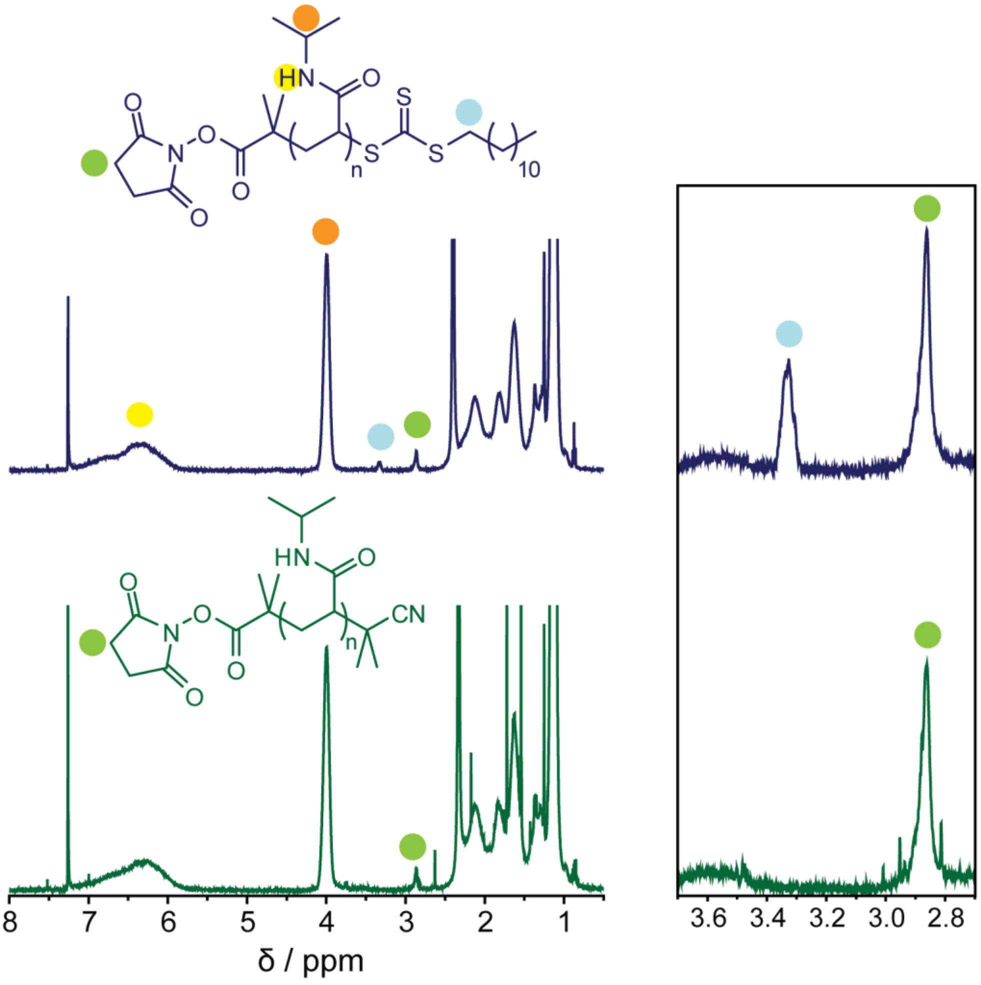

Figure S14 ^{1}H NMR spectrum showing the retention of the NHS activated ester group and the removal of the trithiocarbonate from **P3** upon exposure to AIBN and LPO. Solvent: $CDCl_3$.

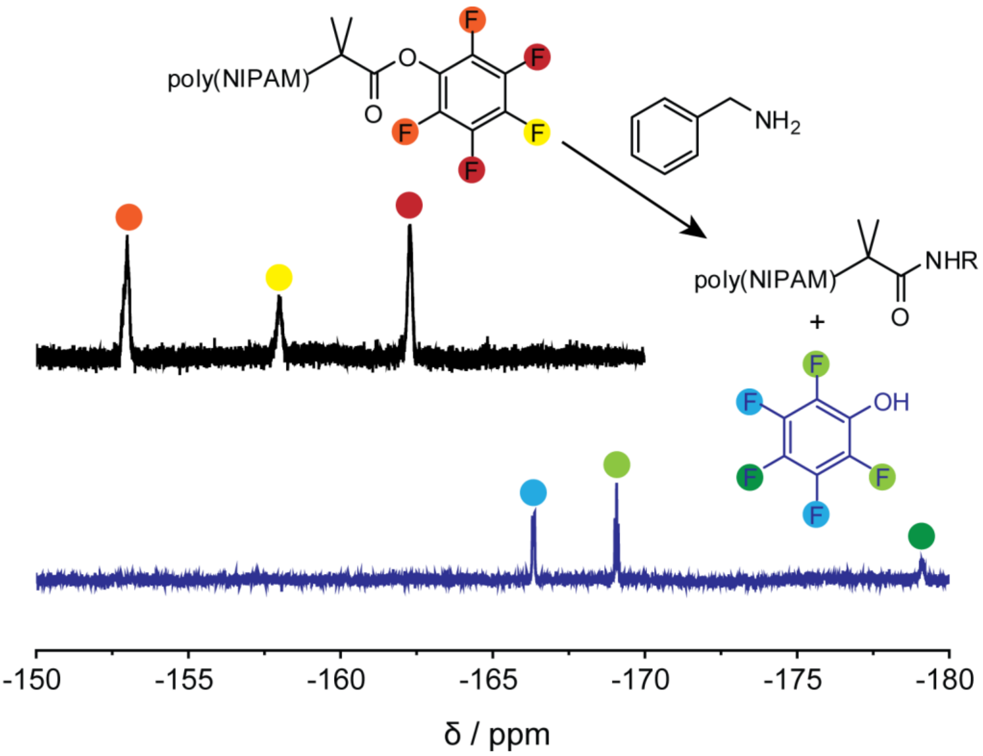

Figure S15 ^{19}F NMR spectrum of **P2**, showing the release of pentafluorophenol upon exposure to benzylamine. R = Bn. Solvent: $CDCl_3$.

Table S4 Reaction conditions tested for the conjugation of **P2** (reactions 5 and 7) and **P3** (reactions 6 and 8) to **s0–NH$_2$**. All experiments were carried out at room temperature overnight. TEA = Triethylamine. DMAc = *N,N*-Dimethylacetamide.

Reaction #	[DNA] / µM	Catalyst	Eq. of polymer	Solvent
**5/6a**				DMF
**5/6b**			100	DMAc
**5/6c**				NMP
**5/6d**				DMF
**5/6e**		-	10	DMAc
**5/6f**				NMP
**5/6g**				DMF
**5/6h**			1	DMAc
**5/6i**	10			NMP
**5/6j**				DMF
**5/6k**			100	DMAc
**5/6l**				NMP
**5/6m**				DMF
**5/6n**		TEA (0.1 M)	10	DMAc
**5/6o**				NMP
**5/6p**				DMF
**5/6q**			1	DMAc
**5/6r**				NMP
**7/8a**				DMF
**7/8b**			10	DMAc
**7/8c**				NMP
**7/8d**		-		DMF
**7/8e**			1	DMAc
**7/8f**				NMP
**7/8g**	100			DMF
**7/8h**			10	DMAc
**7/8i**				NMP
**7/8j**		TEA (0.1 M)		DMF
**7/8k**			1	DMAc
**7/8l**				NMP

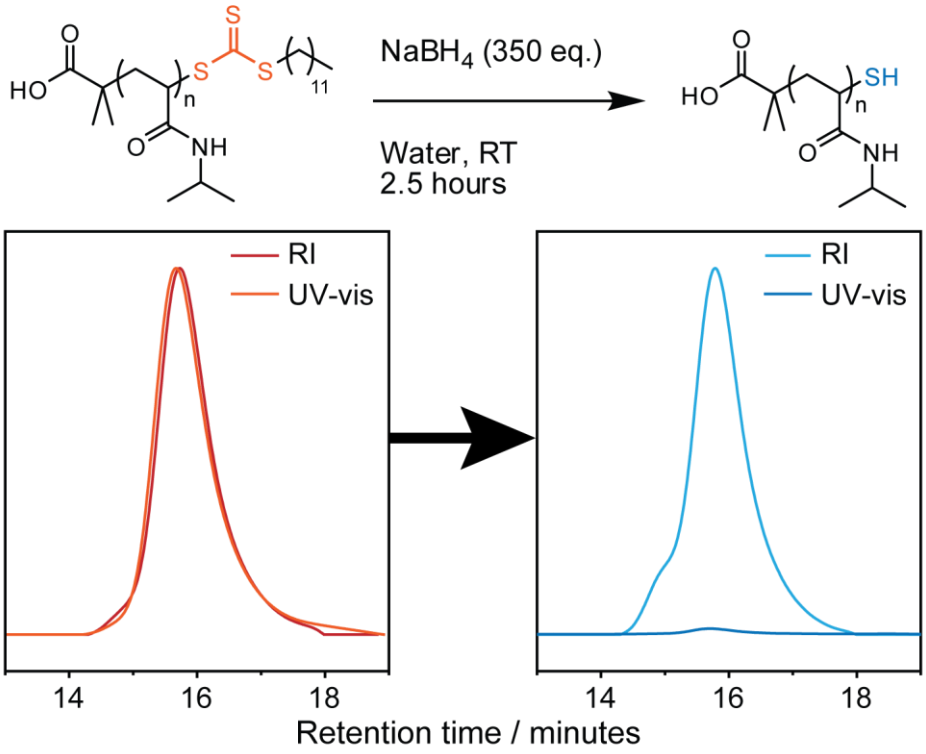

Figure S16 SEC traces showing the removal of the trithiocarbonate group from **P1a-c** using sodium borohydride in water to give **P4a-c**. The refractive index (RI) trace remains essentially unchanged (except for a small high molecular weight shoulder due to disulfide coupling) while the intensity of the UV-vis trace (recorded at 309 nm – the absorbance maximum for the trithiocarbonate) drops almost to zero. RI traces were normalised; UV-vis traces were then scaled by the same amount as their respective RI traces and overlaid.

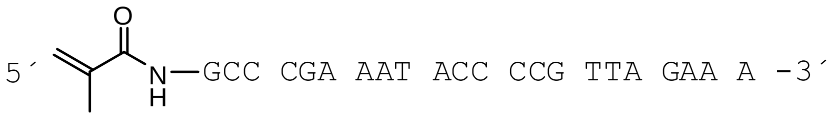

s0-MAAm

Figure S17 Structure of **s0–MAAm**.

Table S5 Reaction conditions tested for the conjugation of **P4a-c** to **s0–MAAm** in the absence of any catalyst. The concentrations of DNA and polymer were kept constant at 10 μM and 1 mM respectively. The reactions were run at 40 °C for twenty-four hours.

Reaction #	Solvent	Polymer
2a		**P4a**
2b	PBS pH 8.0	**P4b**
2c		**P4c**
2d		**P4a**
2e	DMF	**P4b**
2f		**P4c**
2g		**P4a**
2h	DMSO	**P4b**
2i		**P4c**

Table S6 Reaction conditions tested for the conjugation of **P4a-c** to **s0–MAAm** in the presence of TCEP (100 μM). The concentrations of DNA and polymer were kept constant at 10 μM and 1 mM respectively. The reactions were run at 40 °C for twenty-four hours. MOPS = 3-(N-Morpholino)propanesulfonic acid buffer.

Solvent	Polymer
	**P4a**
MOPS pH 8.0	**P4b**
	**P4c**
	**P4a**
DMF	**P4b**
	**P4c**

Table S7 Further reaction conditions trialled for the conjugation of thiol-terminated poly(NIPAM) (**P4a**) to **s0–MAAm**. The DNA concentration was kept constant at 10 µM. Polymer equivalents were measured relative to the methacrylamide group.

Reaction #	Temperature	Solvent	Eq. polymer
5a	RT	H_2O/DMF	1
5b			10
5c			100
5d		DMF	1
5e			10
5f			100
5g		NMP	1
5h			10
5i			100
5j	40°C	H_2O/DMF	1
5k			10
5l			100
5m		DMF	1
5n			10
5o			100
5p		NMP	1
5q			10
5r			100

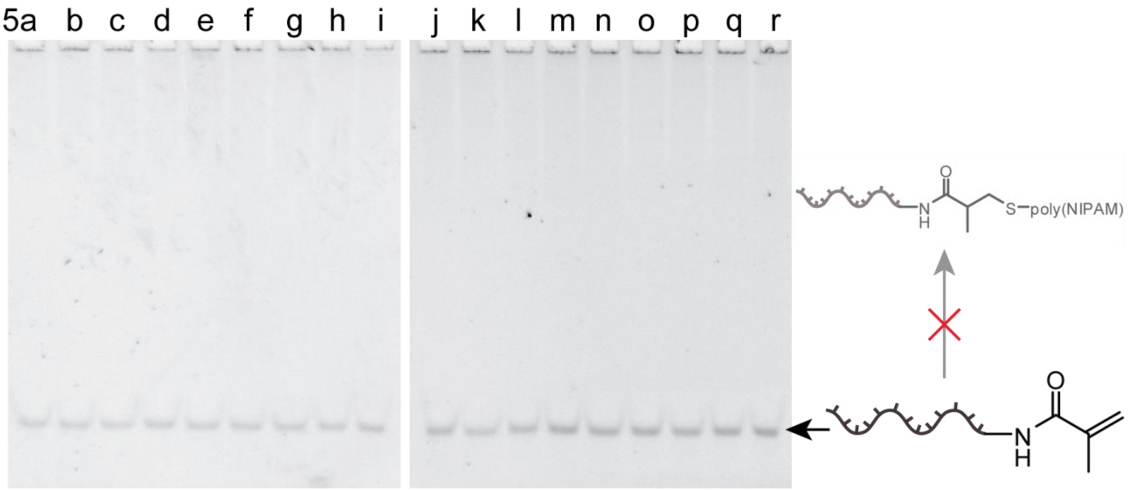

Figure S18 15 % native PAGE analysis of the reaction mixtures detailed in Table S7. The product was expected to appear as a broad, low-mobility band but under no conditions was it observed.

Table S8 Reaction conditions trialled for the conjugation of thiol-terminated poly(NIPAM) (**P4a**) to **s0–MAAm** using DMPP as catalyst. The DNA concentration was kept constant at 10 µM and all reactions were conducted at room temperature under an atmosphere of nitrogen for twenty-four hours.

Reaction #	Solvent	Eq. DMPP	Eq. polymer
6a	DMF	1	10
6b			100
6c		20	10
6d			100
6e	NMP	1	10
6f			100
6g		20	10
6h			100
6i	DMSO	1	10
6j			100
6k		20	10
6l			100

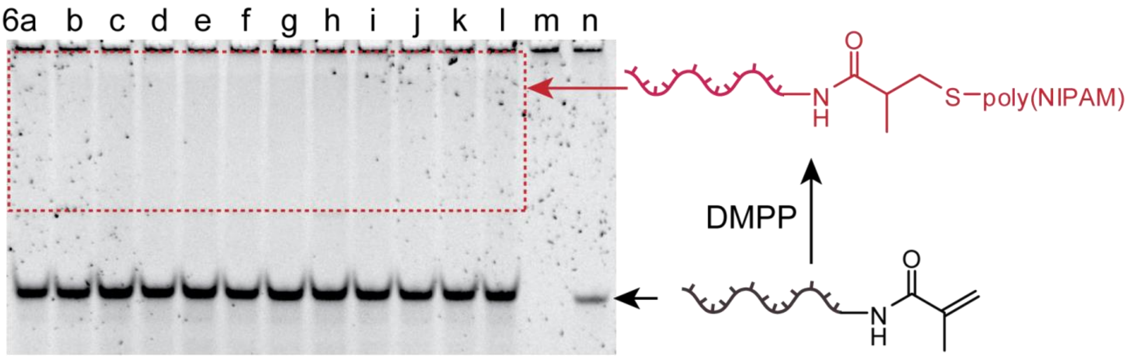

Figure S19 15 % native PAGE analysis of the reaction mixtures detailed in Table S8. A broad, low-mobility band is just visible, possibly indicating the formation of the desired product.

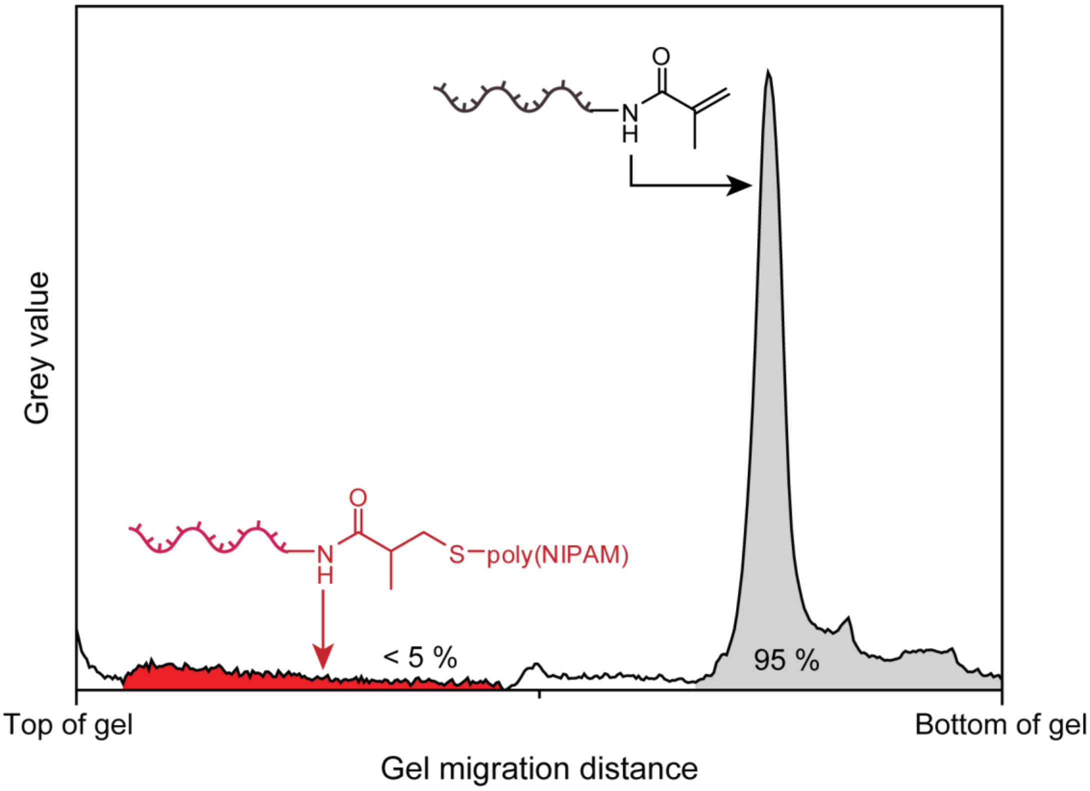

Figure S20 Densitometric analysis of Figure S19, lane 6e. A small hump may be visible at low migration distance, which could be attributed to a very low yield of the DNA–polymer conjugate. Yields (given as percentage values) were estimated by comparing the areas under each peak.

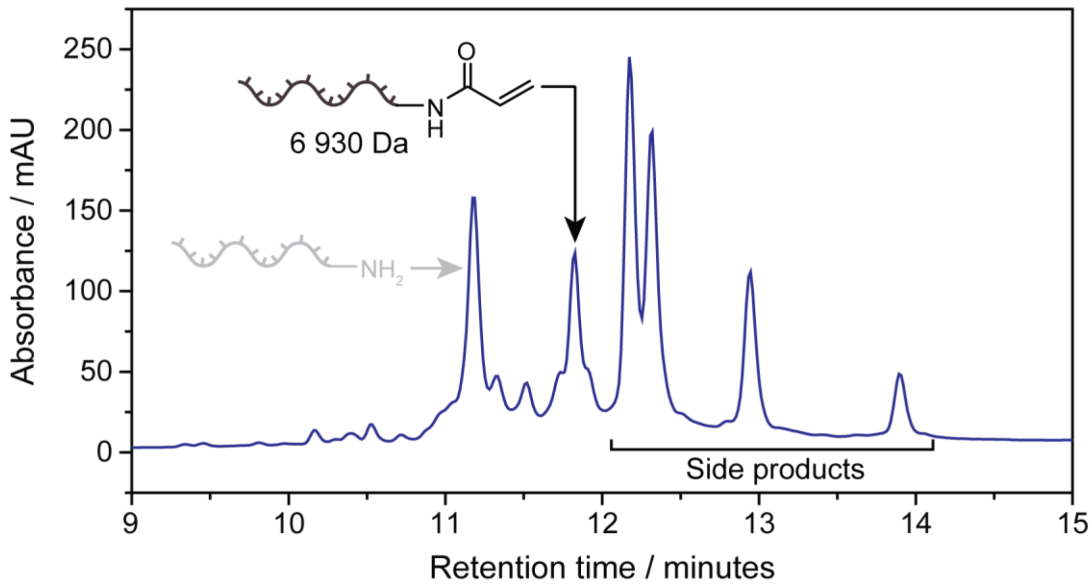

Figure S21 HPLC chromatogram of the reaction mixture during the synthesis of **s0–AAm** (black) from **s0–NH$_2$** (grey). A number of side products were also observed, but were successfully removed by HPLC purification. The product was identified by LC-MS.

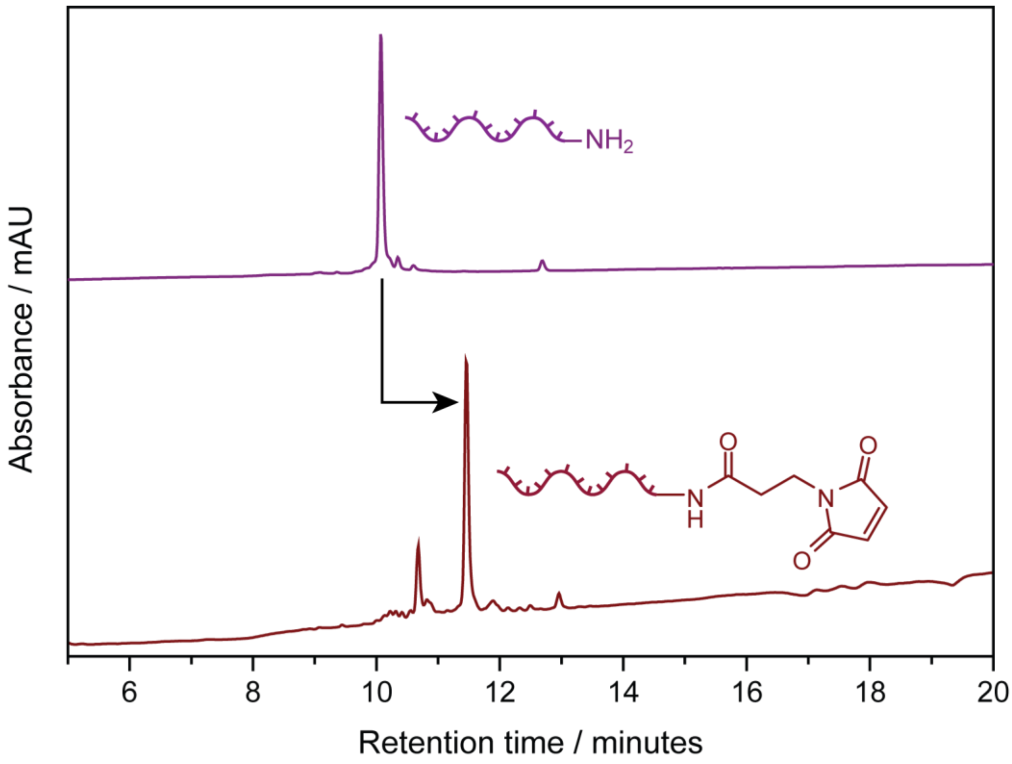

Figure S22 HPLC chromatogram showing the shift in retention time of the DNA peak during the synthesis of **s0–Mal**.

Table S9 Reaction conditions trialled for the conjugation of thiol-terminated poly(NIPAM) (**P4a-c**) to **s0–Mal**, with yields as estimated by PAGE densitometry. All reactions were conducted at 40 °C in MOPS buffer, pH 8. The DNA concentration remained constant at 10 µM.

Reaction #	Polymer (DP)	Polymer eq.	Conjugate yield
7a		2	48 %
7b		5	53 %
7c	**P4a** (50)	25	52 %
7d		100	57 %
7e		500	56 %
7f		2	38 %
7g		5	46 %
7h	**P4b** (97)	25	56 %
7i		100	58 %
7j		500	50 %
7k		2	25 %
7l		5	31 %
7m	**P4c** (196)	25	42 %
7n		100	51 %
7o		500	53 %

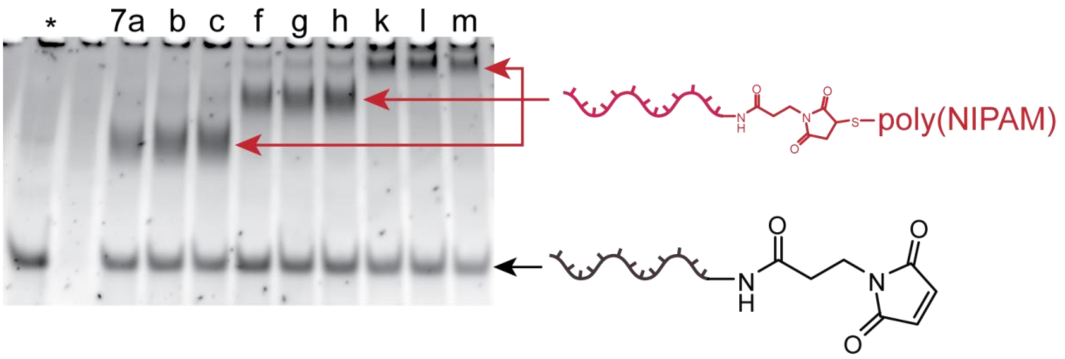

Figure S23 15 % PAGE analysis of the corresponding crude reaction mixtures detailed in Table S9 (for example, lane g corresponds to reaction 7g). Three distinct sets of new bands were observed, corresponding to conjugates containing different molecular weights of poly(NIPAM) (increasing from left to right). Lane * contained the s0–Mal species for comparison.

Table S10 Reaction conditions trialled for the conjugation of thiol-terminated **P4a** with **s0–Mal**. The DNA concentration was 10 μM for all reactions, with a 100-fold excess of polymer and DIPEA (where appropriate). Reactions were performed at 40 °C for sixteen hours.

Reaction #	Solvent	DIPEA?
8a	DMF	
8b	THF	
8c	NMP	No
8d	DMSO	
8e	MeCN	
8f	DMF	
8g	THF	
8h	NMP	Yes
8i	DMSO	
8j	MeCN	

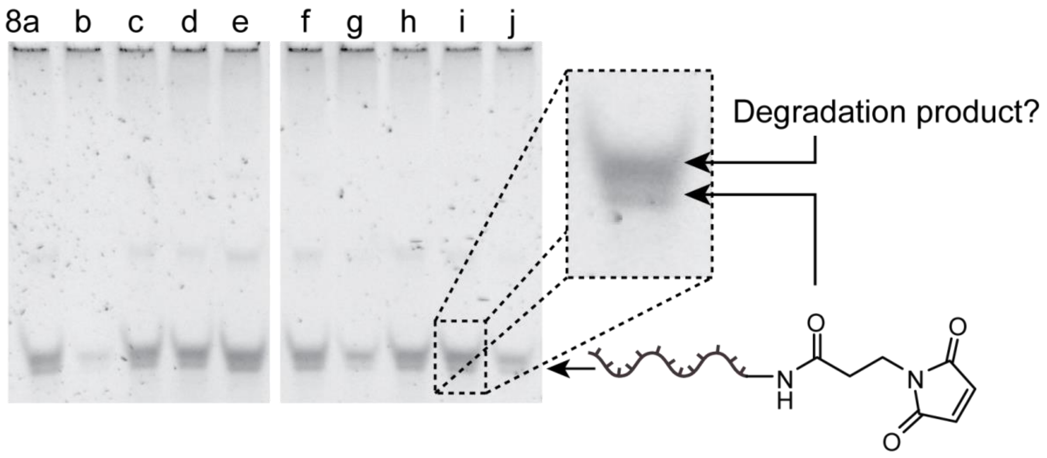

Figure S24 15 % native PAGE analysis of the crude reaction mixtures detailed in Table S10 showing that the conjugation of **P4a** to **s0–Mal** DNA did not work in various organic solvents. Splitting of the band due to the starting material implied degradation of the maleimide group was occurring. Under identical conditions in MOPS buffer the conjugate was produced in around 50 % yield.

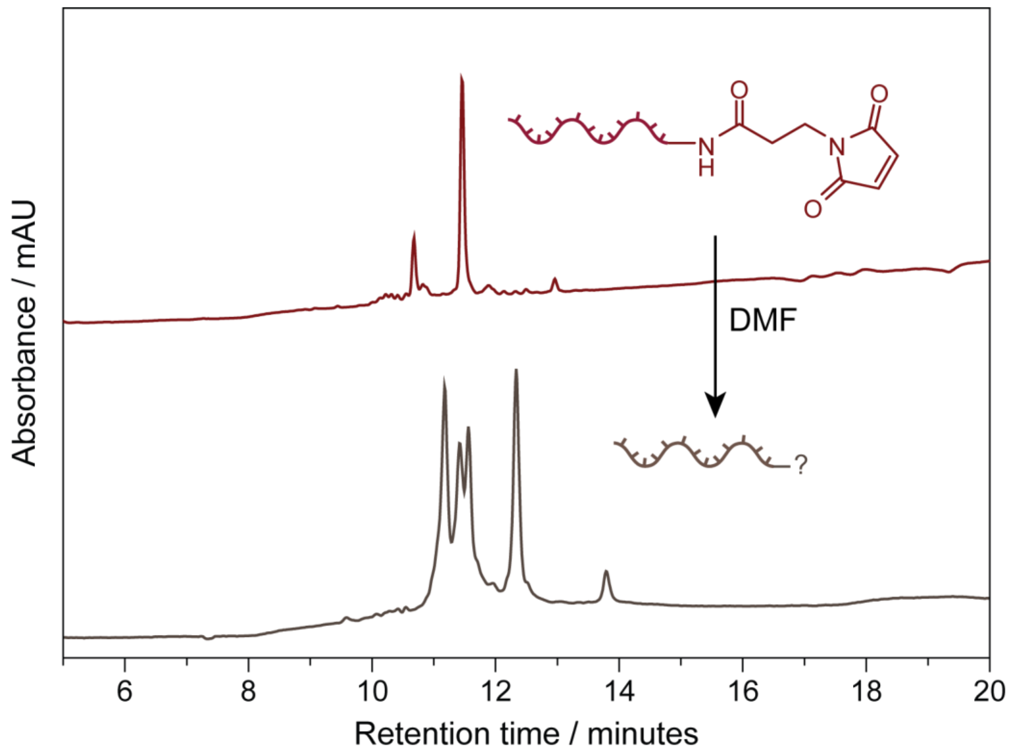

Figure S25 HPLC chromatograms of **s0–Mal** DNA before (top, red) and after (bottom, brown) incubation in DMF for 24 hours.

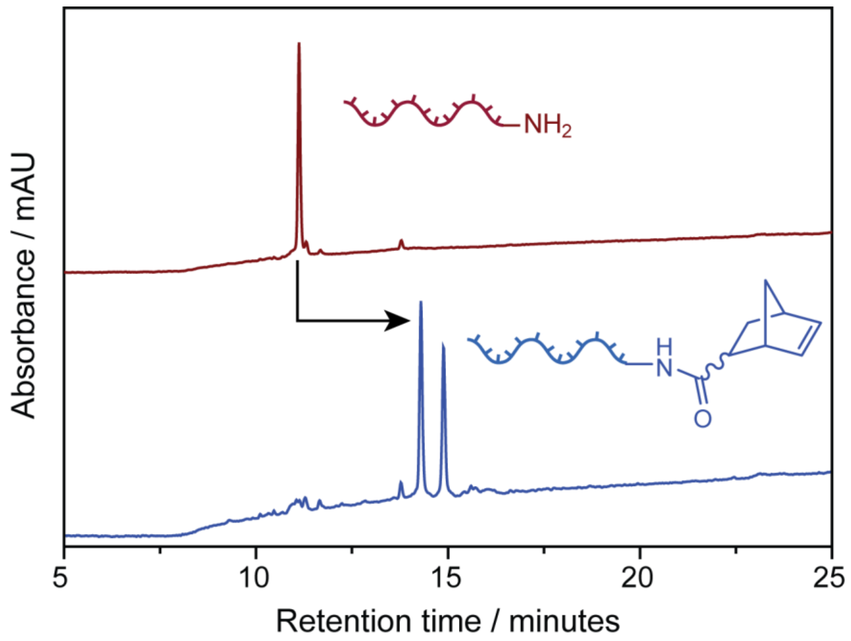

Figure S26 HPLC chromatograms showing the formation of **s0–Nb** (bottom) from **s0–NH$_2$** (top) and a Nb acid.

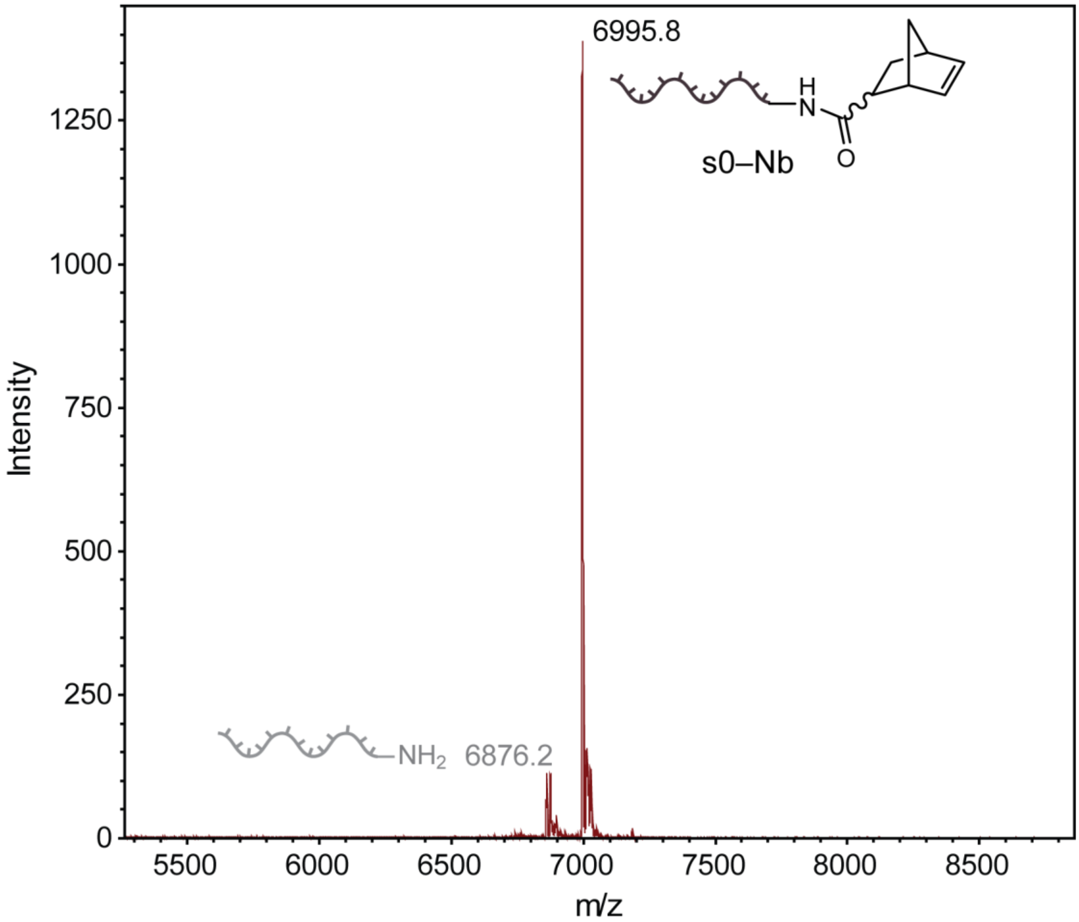

Figure S27 MALDI-ToF mass spectrum of **s0–Nb** using 3-Hydroxypicolinic acid (3-HPA) as matrix. A small amount of the **s0–NH$_2$** starting material was observed. The expected peak molecular weight of the product was 6996.3 Da.

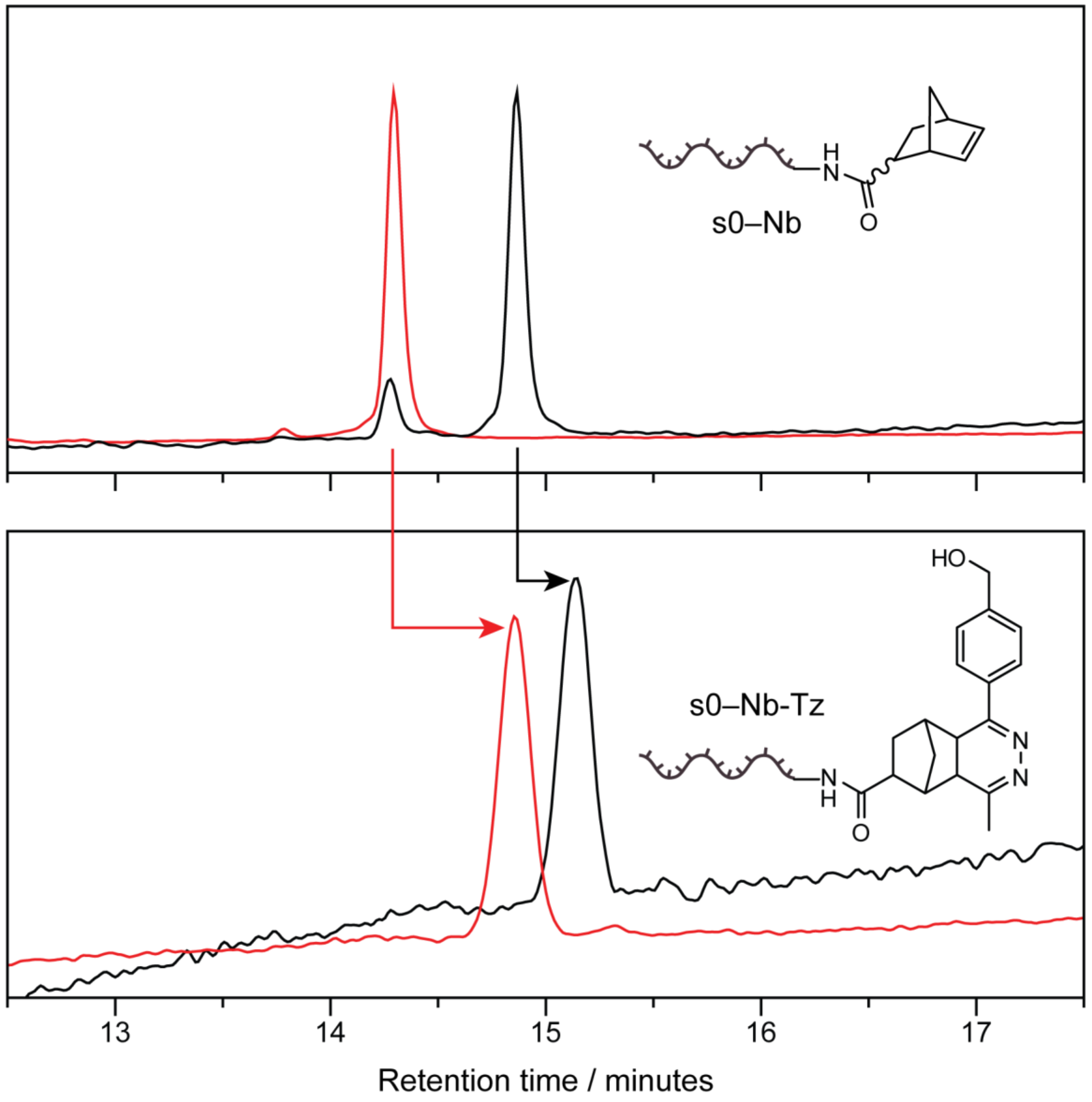

Figure S28 HPLC analysis of the reaction of **s0–Nb** with the small molecule Tz **6**. A clear peak shift was observed for both the endo and exo isomers, indicative of a successful reaction. Reaction conditions: **s0–Nb** (10 μM), **6** (1 mM), HPLC buffer (100 mM TEAA, 70 % MeCN), room temperature, 24 hours.

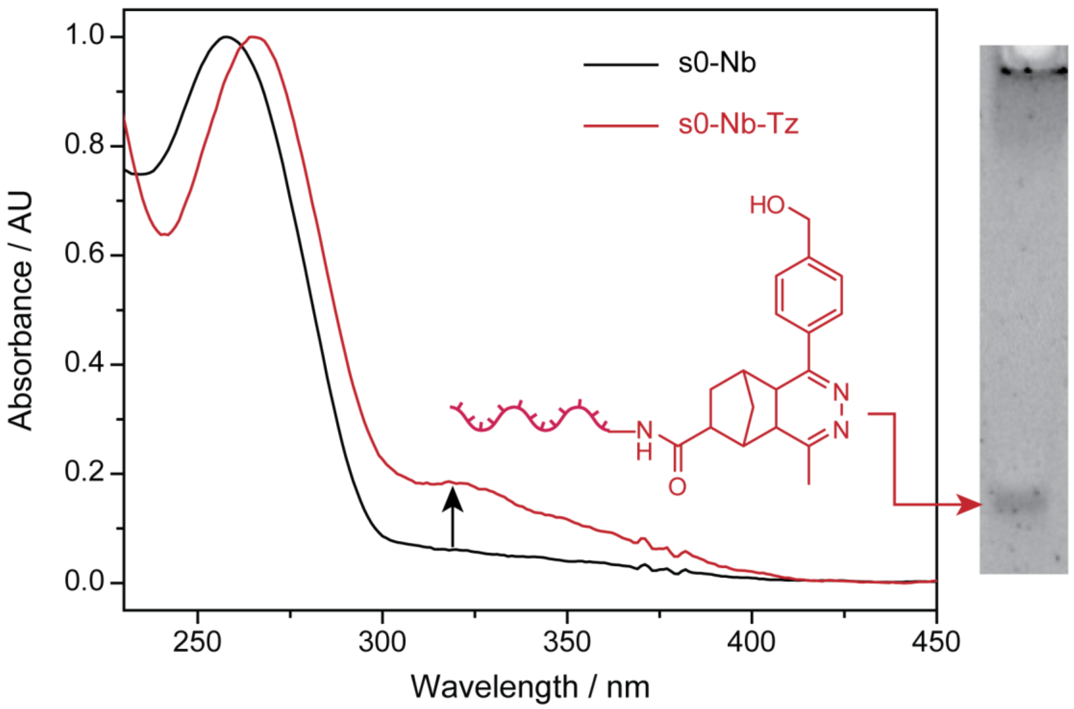

Figure S29 UV-vis spectra showing the change in absorbance maxima when the Tz- containing alcohol **6** was added to **s0–Nb** DNA. Addition of the Tz caused a shift in the principal peak from 260 nm to 265 nm, and a new peak was observed at around 310 nm. 15 % native PAGE (right), stained with a nucleic acid-specific dye, was used to confirm the presence of DNA in the sample.

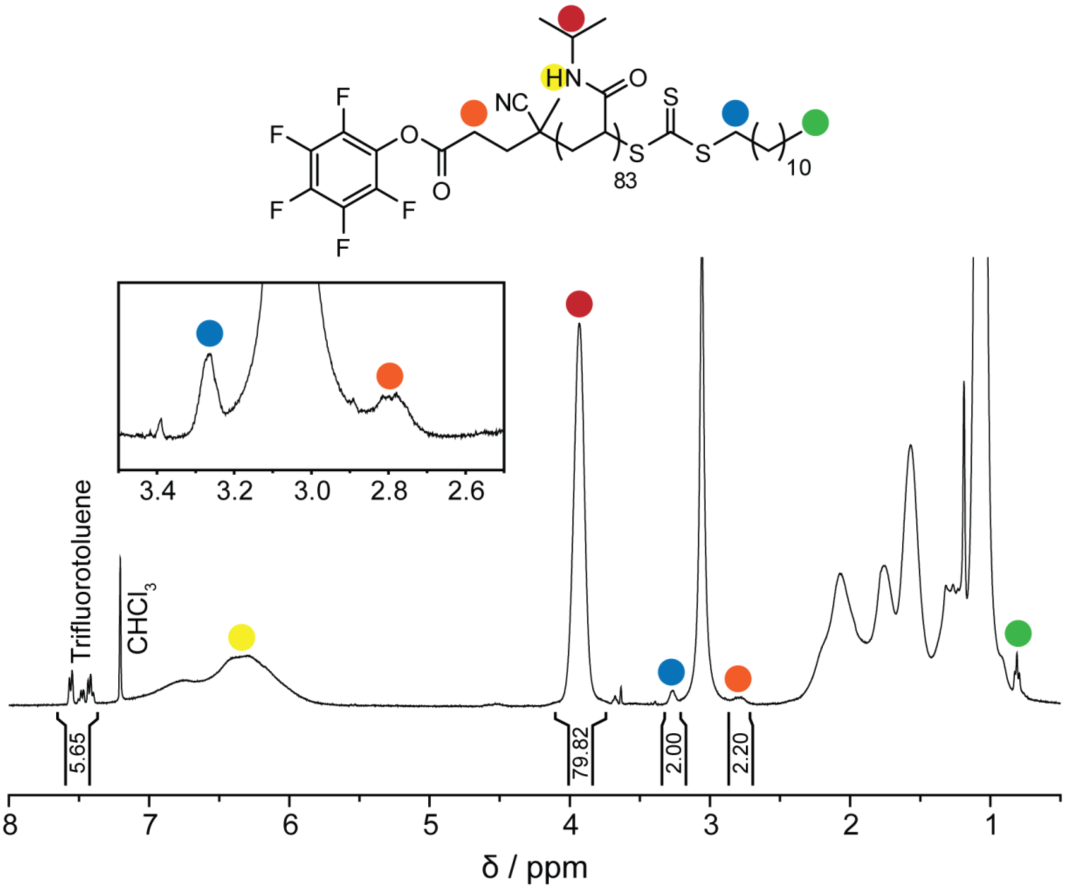

Figure S30 ^{1}H NMR spectrum of **P6** showing the presence of the key peaks due to the polymer end groups. Trifluorotoluene was included as an external standard to assess the degree of incorporation of the PFP activated ester group. Solvent: $CDCl_3$.

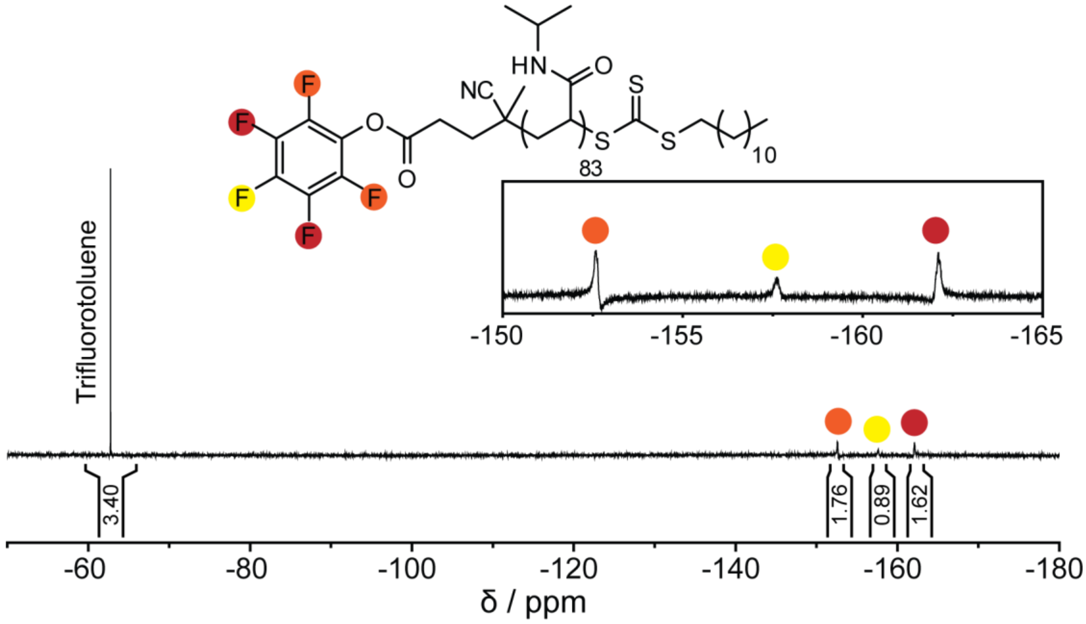

Figure S31 ^{19}F NMR spectrum of **P6** showing the presence of the PFP activated ester peaks. Trifluorotoluene was included as an external standard to assess the degree of incorporation of the PFP activated ester group. Solvent: $CDCl_3$.

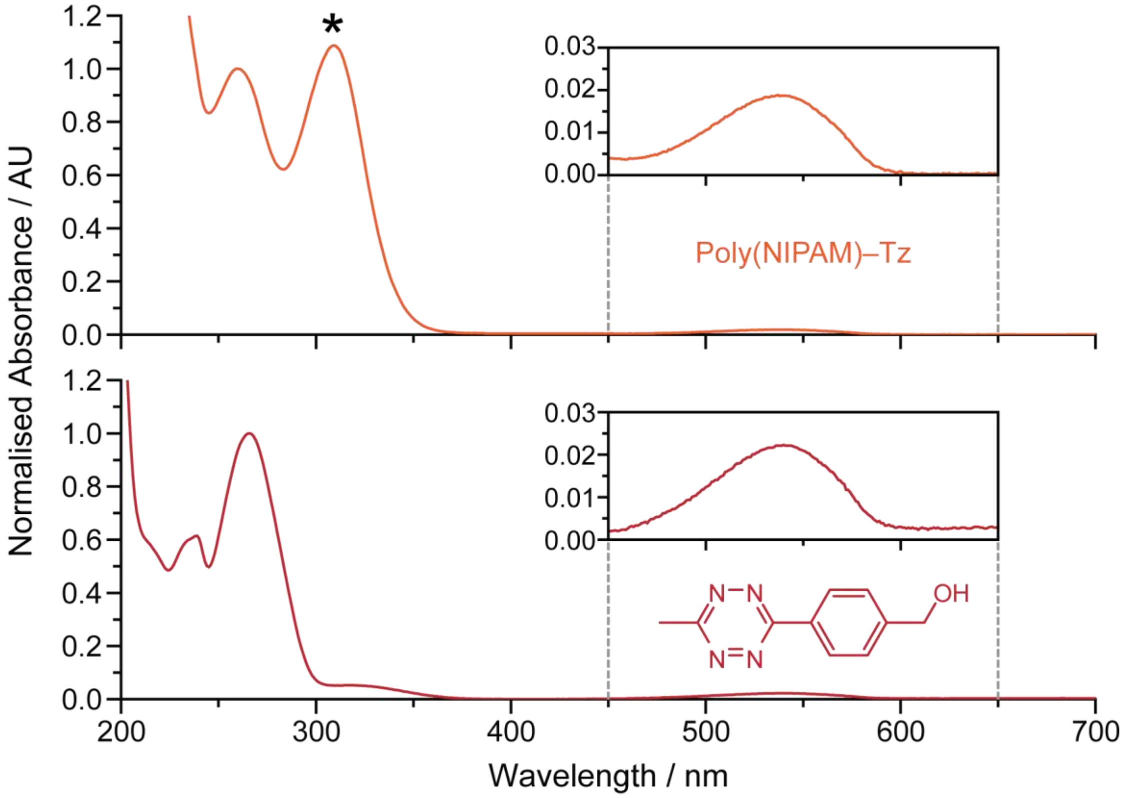

Figure S32 UV-vis spectrum of the tetrazine alcohol **6** (bottom, red) and **P7** (top, orange). The polymer exhibited the characteristic Tz absorbances at 265 nm and 540 nm (insets), as well as a peak at 309 nm due to the trithiocarbonate group at the opposite end of the polymer chain (*).

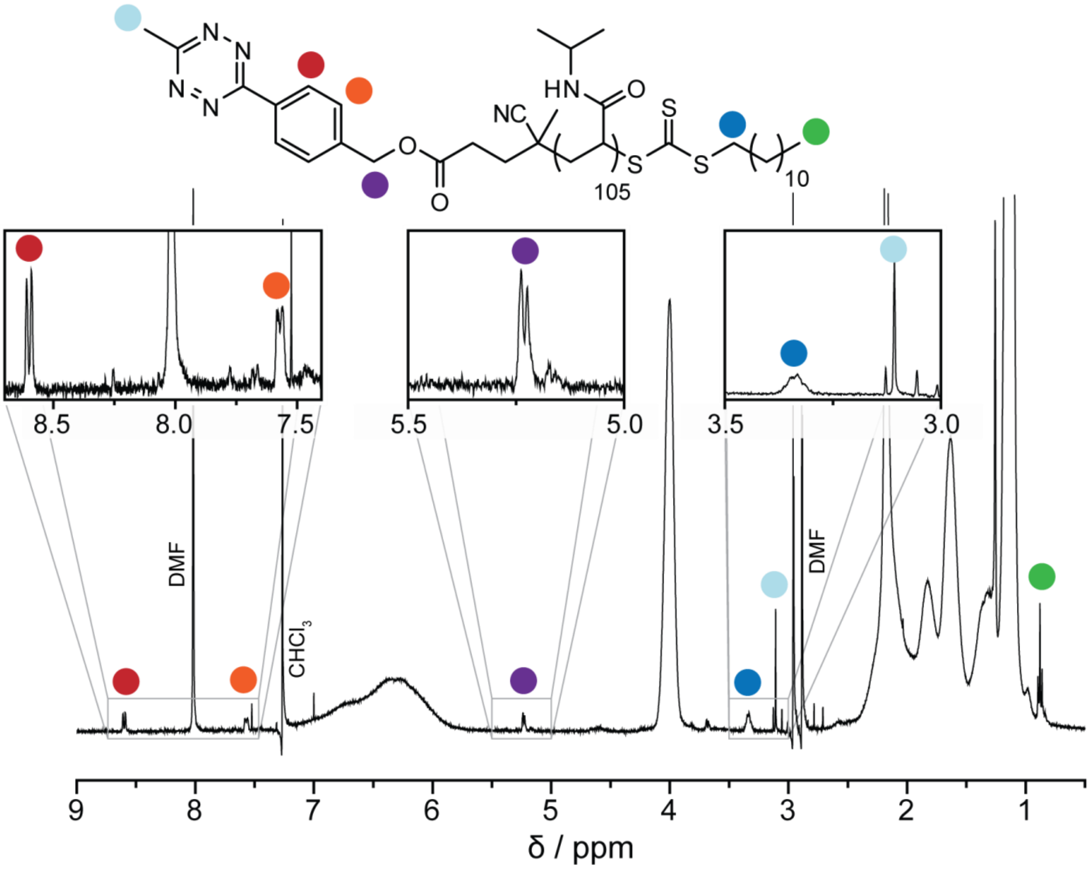

Figure S33 ^{1}H NMR spectrum of **P7**. The peaks due to both end groups are clearly visible, and all the peaks due to the Tz group are accounted for. Integration of the Tz signals to the poly(NIPAM) NHCH peak at 4.00 ppm revealed that this group had been incorporated with an efficiency of 53 %. Solvent: $CDCl_3$.

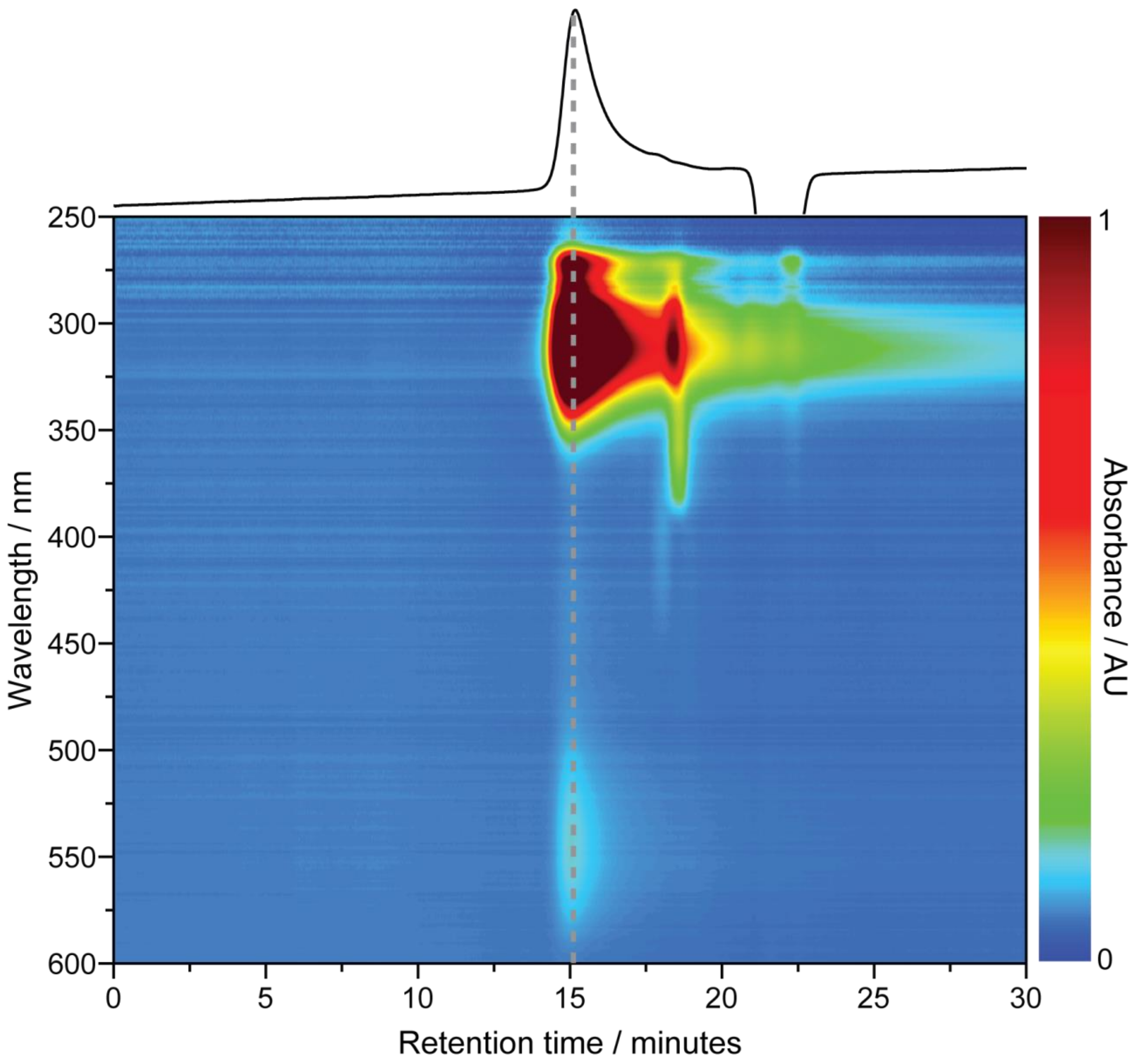

Figure S34 THF SEC refractive index chromatogram (top) and UV-vis 2D colour map (bottom) of **P7**. The colour map clearly shows that the characteristic Tz peak at 540 nm is only associated with the polymer, confirming its successful incorporation at the chain end.

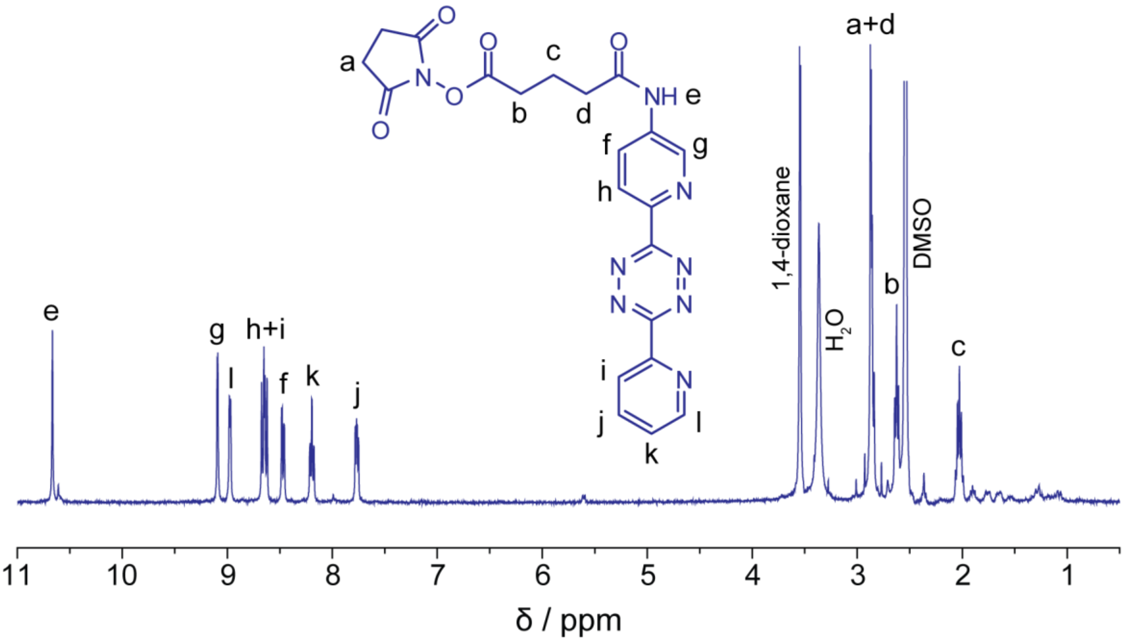

Figure S35 ^{1}H NMR spectrum of the Tz-containing activated ester, **10**. Solvent: d_6-DMSO.

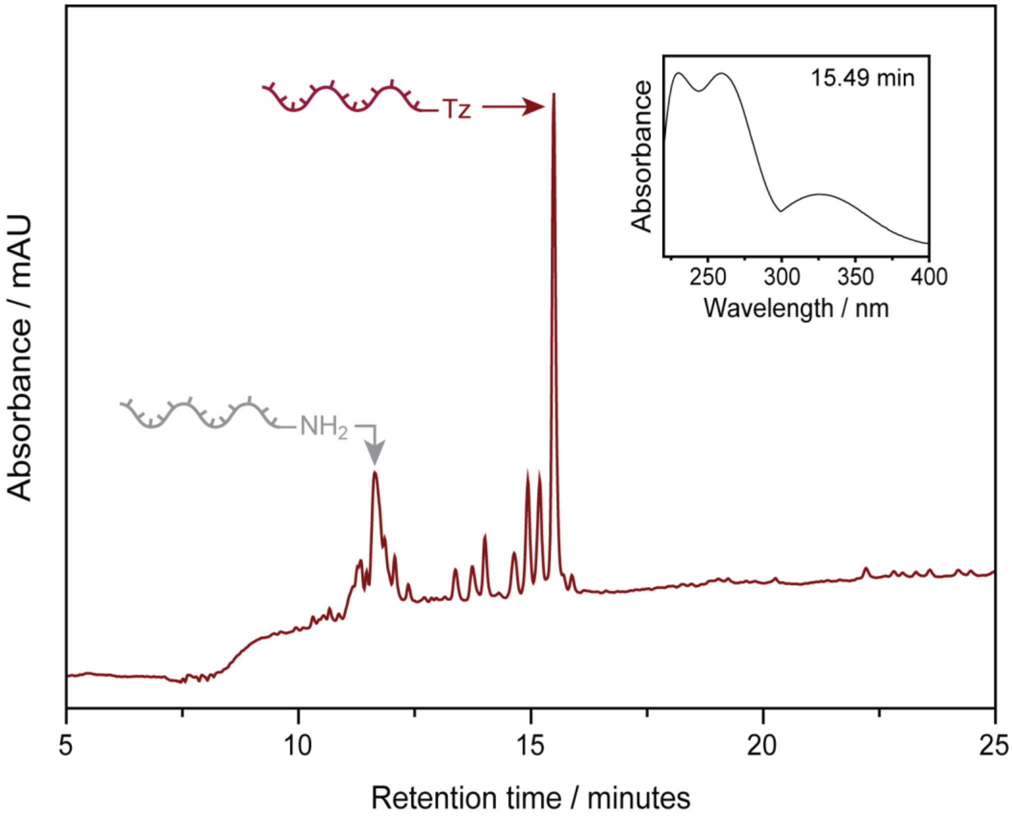

Figure S36 HPLC chromatogram showing the formation of **s0–Tz**. The UV-vis spectrum (inset) of the indicated peak exhibits a maximum at around 330 nm – this is characteristic of the Tz group.

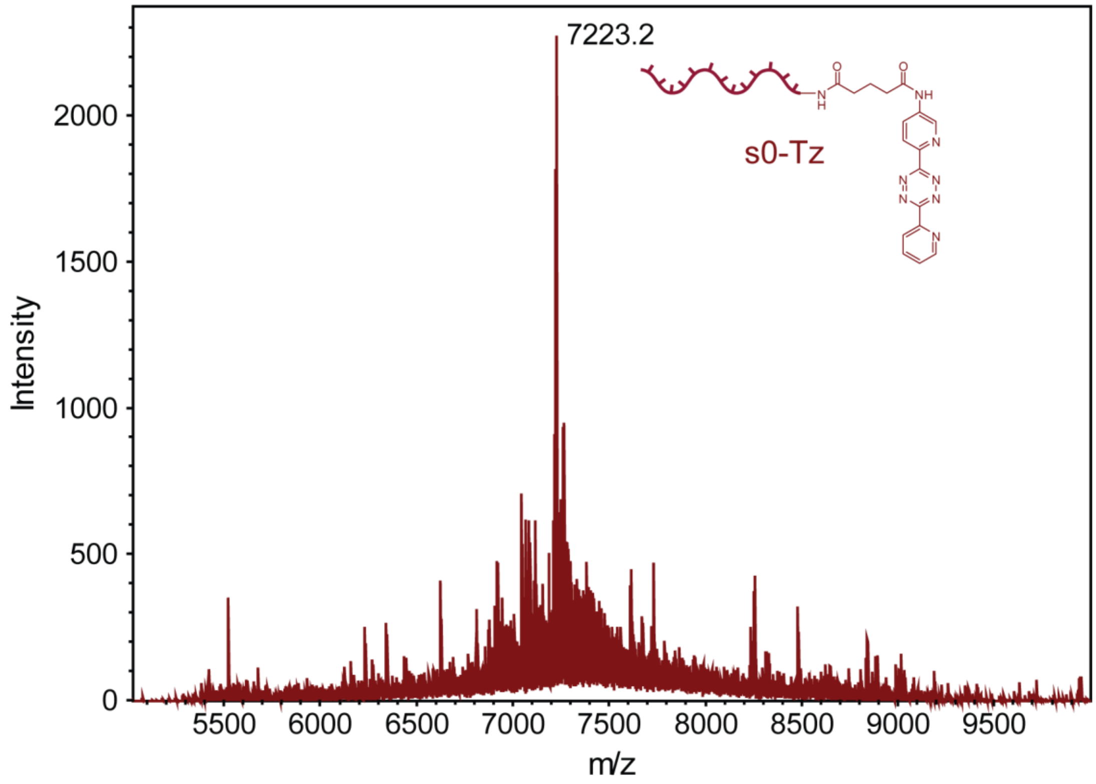

Figure S37 ESI MS spectrum of the **s0–Tz** molecule. The expected mass was 7 223.9 Da.

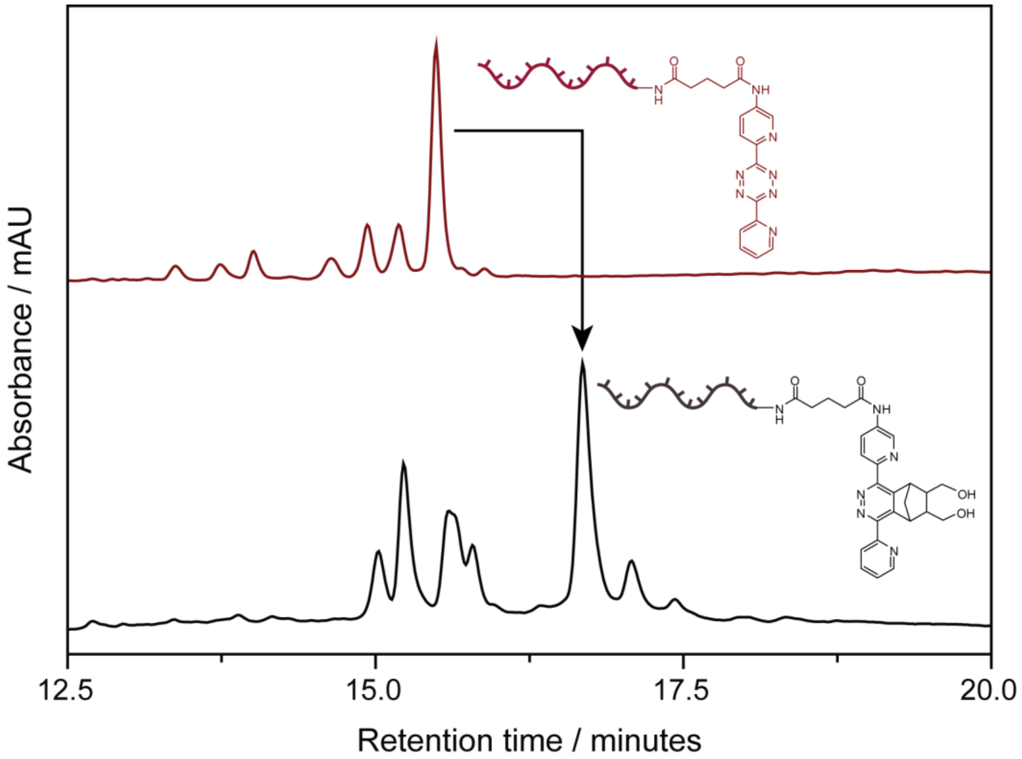

Figure S38 HPLC chromatograms showing the shift in retention time that occurred for the principal peak when a small molecule norbornene alcohol was added to **s0–Tz**.

3 References

(1) Skey, J.; O'Reilly, R. K. *Chem. Commun.* **2008**, 4183.

(2) Hansell, C. F.; Espeel, P.; Stamenović, M. M.; Barker, I. A.; Dove, A. P.; Du Prez, F. E.; O'Reilly, R. K. *J. Am. Chem. Soc.* **2011**, *133*, 13828.

(3) Hansell, C. F.; O'Reilly, R. K. *ACS Macro Lett.* **2012**, *1*, 896.

(4) Panne, P.; Fox, J. M. *J. Am. Chem. Soc.* **2006**, *129*, 22.
